# A 2D-proteomic analysis identifies proteins differentially regulated by two different dengue virus serotypes

**DOI:** 10.1038/s41598-024-57930-1

**Published:** 2024-04-09

**Authors:** Chanida Chumchanchira, Suwipa Ramphan, Atchara Paemanee, Sittiruk Roytrakul, Pathrapol Lithanatudom, Duncan R. Smith

**Affiliations:** 1 PhD Degree Program in Biology (International Program), Department of Biology, Faculty of Science, Chiang Mai University, Chiang Mai, 50200 Thailand; 2 Institute of Molecular Biosciences, Mahidol University, Nakhon Pathom, 73170 Thailand; 3 National Center for Genetic Engineering and Biotechnology (BIOTEC), National Science and Technology Development Agency, Khlong Luang, Pathum Thani 12120 Thailand; 4 Department of Biology, Faculty of Science, Chiang Mai University, Chiang Mai, 50200 Thailand

**Keywords:** Virology, Cellular microbiology

## Abstract

The mosquito transmitted dengue virus (DENV) is a major public health problem in many tropical and sub-tropical countries around the world. Both vaccine development and drug development are complex as the species *Dengue virus* consist of four distinct viruses (DENV 1 to DENV 4) each of which is composed of multiple lineages and strains. To understand the interaction of DENV with the host cell machinery, several studies have undertaken in vitro proteomic analysis of different cell lines infected with DENV. Invariably, these studies have utilized DENV 2. In this study we sought to define proteins that are differentially regulated by two different DENVs, DENV 2 and DENV 4. A 2-dimensional proteomic analysis identified some 300 protein spots, of which only 11 showed differential expression by both DENVs. Of these, only six were coordinately regulated. One protein, prohibitin 1 (PHB1) was downregulated by infection with both DENVs. Overexpression of PHB1 increased DENV protein expression, level of infection and genome copy number. DENV E protein colocalized with PHB, and there was a direct interaction between DENV 2 E protein and PHB1, but not between DENV 4 E protein and PHB1. The low number of proteins showing coordinate regulation after infection by different DENVs is a cause for concern, particularly in determining new druggable targets, and suggests that studies should routinely investigate multiple DENVs.

## Introduction

The genus *Flavivirus* contains some 52 viral species^1^ of which the species *Dengue virus* causes the greatest impact on human health around much of the tropical and sub-tropical world^2^. The species *Dengue virus* consists of four viruses, dengue virus (DENV) 1, DENV 2, DENV 3 and DENV 4, which share a high degree (about 65–70%) of sequence similarity^3^, showing that these viruses are closely related, but they are antigenically distinct^4^. DENV is a mosquito-borne virus, mainly transmitted by *Aedes* (*Ae*) species mosquitoes, especially *Ae. aegypti* and *Ae. albopictus*^5^. Annually, about 400 million people are infected, albeit that 80% of these infections are asymptomatic^2^. Symptomatic infection can range from a mild undifferentiated fever, to dengue fever hallmarked by fever, rash, headache and muscle and joint pains to the more severe presentations of dengue hemorrhagic fever and dengue shock syndrome which are primarily hallmarked by severe hemorrhage^5^. Annually, some 40,000 deaths are believed to occur as a result of DENV infection^2^.

DENV is a positive-sense single stranded RNA virus with a genome of approximately 11 kb encoding for one open reading frame which is directly translated into a polyprotein composed of three structural proteins (envelope (E), precursor of membrane (prM, which is cleaved to generate M during virion maturation) and capsid (C)) and seven non-structural (NS) proteins (NS1, NS2A, NS2B, NS3, NS4A, NS4B, and NS5^6^. The seven non-structural proteins form the replication complex which produces an intermediate negative sense RNA that is used as a template to generate new positive sense genomes (reviewed in^7^). In addition to forming the replication complex, the non-structural proteins play critical roles in modulating the host cell to dampen the innate immune system^8^, and to modify the cellular proteome^9–14^ and lipidome^15^ to generate a cellular environment that favors viral replication.

To undertake infection of a susceptible and permissive host cell, the DENV virion binds to host cell surface receptors and enters predominantly through clathrin-mediated endocytosis^16^. Due to acidic pH changes during endosomal trafficking, the virus fuses to the endosome resulting release of the nucleocapsid in to the cytoplasm. After uncoating of the RNA genome it is directly translated into a single polypeptide that is processed by virally encoded and host cell proteases. Viral replication occurs in replication vesicles closely associate with the endoplasmic reticulum, following which the immature virion is trafficked to the Golgi body where virion maturation occurs as a consequence of cleavage of prM to M, mediated by host cell expressed furin (reviewed in^7^).

A number of studies have attempted to understand the processes that mediate changes induced by DENV infection by looking at cellular protein changes induced by DENV infection through proteomic analysis of a number of different cell lines^9–14^. One of the previous studies was a combined proteome and phosphoproteome analysis^11^, and one other study conducted a phosphoproteome analysis^17^. Invariably, these studies utilized DENV 2 as the infecting virus. However, in a previous study utilizing 12 DENV viruses^18^ representing the four different DENV viruses as well as representing high passage strains, and low passage strains isolated from dengue fever (DF) or dengue hemorrhagic fever (DHF) patients we showed using MALDI-TOFF and PCA analysis that there was no clear segregation of the data by either virus (DENV 1, DENV 2, DENV 3 and DENV 4) or virus origin (high passage, low passage isolates from DF or DHF patients). The study further undertook a LC–MS/MS analysis on pooled sub-10 kDa fractions and identified 128 proteins, of which only 20% were consistently expressed in all samples, while the remaining 80% were variably expressed^18^. This suggested that there is a high degree of plasticity in how DENV interacts with and alters the cellular proteome. Given that the previous studies have exclusively looked at DENV 2, this study attempted to determine how many proteins were commonly differentially expressed in response to DENV 2 or DENV 4 infection.

## Materials and methods

### Cells and viruses

HEK293T/17 (human embryonic kidney) cells were cultured in Dulbecco’s minimal essential medium (DMEM, Gibco, Invitrogen, Carlsbad, CA) supplemented with 10% heat-inactivated fetal bovine serum (FBS, Gibco, Invitrogen) and incubated at 37 °C with 5% CO_2_.

High passage Dengue virus serotype 2 (DENV 2; strain 16681 also known as strain Thailand/16681/1984; GenBank MK506263) and low passage dengue virus serotype 4 (DENV 4; strain ss14/163; GenBank MK301275.1) isolated from a patient with undifferentiated fever were propagated in C6/36 (*Aedes albopictus*) cells as previously described^19^. After virus propagation the cell supernatant was centrifuged at 1000xg to remove cell debris and the supernatant was then supplemented with 20% heat-inactivated FBS (Gibco, Invitrogen) and stored frozen at − 80 °C. Virus titer was determined by plaque assay on LLC-MK2 (Rhesus monkey kidney) cells as described previously^20^.

### Virus infection

HEK293T/17 cells were seeded in six-well plates and cultured under standard growth condition for 24 h. After the cells reached ~ 70–80% confluence, the cell culture medium was removed, and the cells were either mock infected or infected with either DENV 2 or DENV 4 at a multiplicity of infection (MOI) of 5 for 2 h. Subsequently, the virus containing medium was replaced by fresh culture medium and cells were further incubated under standard conditions until an appropriate time point was reached. All experiments were undertaken as three independent biological replicates.

### Flow cytometry

Mock-, DENV 2 or DENV 4 infected cells were harvested at the indicated time point and blocked with 10% normal goat serum (Gibco, Invitrogen) on ice for 30 min. After that, the cells were fixed with 4% paraformaldyhyde and permeabilized using 0.2% of Triton X-100 as described previously^21^. Subsequently, the cells were incubated overnight at 4 °C with a pan-specific mouse anti-dengue virus E protein monoclonal antibody produced in house from hybridoma HB114^22^ diluted 1:150. After washing with 1% BSA in PBS-IFA, the cells were incubated in the dark with a 1:40 dilution of a FITC-conjugated goat anti-mouse IgG (sc2010; Santa Cruz Biotechnology Inc., Dallas, TX) for 1 h. Samples were analysed on a BD FACSCalibur cytometer (BD, Franklin Lakes, NJ) using CELLQuest software (version 3.3). All experiments were undertaken independently in triplicate. See Supplementary Table S4 for antibody details.

### Two-dimensional (2D)-gel electrophoresis

The cell pellets from mock- and DENV-infected HEK293T/17 cells were lysed by addition of RIPA buffer (1% NP-40, 0.5% sodium deoxycholate, 0.1% sodium dodecyl sulfate, 137 mM sodium chloride, 2.7 mM potassium chloride, 4.3 mM disodium hydrogen phosphate, 1.4 mM potassium dihydrogen phosphate) containing a protein inhibitor cocktail (PIC), and proteins were precipitated overnight by addition of acetone and methanol. Subsequently the protein pellets were dissolved in DTT and lysis C buffer (8 M urea, 2 M thiourea, 4% CHAPS, 20 mM DTT, 1 mM PMSF, 1 mM benzamide) was added prior to determination of the protein concentration by the Bradford assay. Subsequently, 250 µg of purified proteins were loaded onto Immobiline Drystrips (pH 3–10 NL, 7 cm) containing 2% IPG buffer (Amersham Biosciences) and 0.5% bromophenol blue and the strips were rehydrated for 12 h. Proteins were subjected to isoelectric focusing with a Multipor II electrophoresis system (Amersham Biosciences) at the following voltages: 300 V for 200 Vh, 1000 V for 300 Vh, a gradient to 3000 V for 4000 Vh, 5000 V for 4500 Vh and 5000 V for 3000 Vh. After focusing, the IPG strips were reduced in equilibration buffer (50 mM Tris–HCl (pH 8.8), 6 M urea, 30% v/v glycerol, 2% SDS w/v and 1% bromophenol blue) supplemented with 100 mM DTT for 15 min and then alkylated in equilibration buffer containing 150 mM iodoacetamide (IAA) for 30 min. The proteins were separated in the second dimension via 12.5% SDS-PAGE. Gels were stained with Coomassie Blue G250 after which the gels were visualized under an appropriate detection system. All treatments were performed as three independent biological replicates. Image data were analyzed using ImageMasterTM 2D Platinum version 7.0 software (Amersham Biosciences). Statistical analysis was performed by student’s t test with a p value of less than 0.05 being considered as statistically significant.

### Tryptic in gel digestion and protein identification by LC/MS/MS

Consistently differentially expressed protein spots were cut from the gels and subjected to in-gel tryptic digestion according to the method described elsewhere^23,24^. Peptide mixtures were analyzed by an ultra-performance liquid chromatography (UPLC) (Ultimate 3000, Dionex, Sunnyvale, CA) coupled to a microTOF-Q II™ ESI-Qq-TOF mass spectrometer (Bruker Daltonics, Germany). The MS/MS spectra produced from each sample were searched against the NCBI databases using the MASCOT search engine (Matrix Science, London, United Kingdom).

### Western blot assay

Purified protein samples from mock- and DENV infection of HEK293T/17 cells were separated by 12% SDS-PAGE and subsequently transferred to nitrocellulose membranes. The protein containing membranes were probed with antibodies directed against heat shock protein 70 1A (HSPA1A), heat shock protein 90 (HSP90), stress-induced phosphoprotein 1 (STIP1), prohibitin 1 (PHB1) and DENV E protein (HB112)^22^ followed by an appropriate HRP conjugated secondary antibody at the desired dilution (Supplemental Table S1).

### PHB1 expression plasmid construction

RNA was extracted from HEK293T/17 cells using Trizol reagent (Invitrogen, Thermo Fisher, Waltham, MA) following the manufacturer’s instructions. cDNA was synthesized from 3 µg of RNA using oligo(dT)_17_ primers (Invitrogen) and incubation at 94 °C for 2 min followed by adding RevertAid Reverse Transcriptase (Thermo Fisher) and subsequently following the cycle conditions according to the manufacturer’s protocol. To generate a PHB1 clone (GenBank accession number BT007411.1) the cDNA was used as a template for amplification with specific primers for PHB1 (PHB1_F: 5′-AAGCTTgccaccATGGCTGCCAAAG-3′; PHB1_R: 5′-GAATTCCTACTGGGGCAGCTGG-3′) using Phusion High-Fidelity DNA polymerase (Thermo Fisher). The PCR cycles conditions were 98 °C for 30 s, followed by 30 cycles of 98 °C for 10 s, 58 °C for 30 s and 72 °C for 30 s, followed by a final extension of 72 °C for 5 min. To generate a clone of PHB1, the PCR products were digested with restriction enzymes NheI and BamHI (Thermo Fisher), then ligated into pCDH-EF1-FHC expression vector which contains a HA-tag (Addgene, Watertown, MA), prior to transformation into *Escherichia coli* DH5α competent cells. The bacteria colonies were collected and the DNA plasmids extracted using a GF-1 plasmid DNA extraction kit (Vivantis, Selangor, Malaysia) following the manufacturer’s instruction. Colony PCR was performed using DreamTaq™ DNA polymerase (Thermo Fisher) with PHB1 primers; the PCR condition for which were 95 °C for 3 min, followed by 35 cycles of 95 °C for 30 s, 52 °C for 30 s and 72 °C for 1 min, followed by a final extension of 72 °C for 5 min. Putative positive clones were confirmed by commercial DNA sequencing (Apical Scientific, Selangor, Malaysia).

### Plasmid transfection

HEK293T/17 cells were seeded into six-well tissue culture plates at a density that allowed approximately 60% confluence to be reached on the day of transfection. The cells were transfected with a eukaryotic expression plasmid containing human prohibitin 1 (pCDH-EF1-FHC_PHB1) in parallel with the empty plasmid vector as a negative control, using Lipofectamin 3000 (Invitrogen, Carlsbad, CA) according to the manufacturer's instruction. Briefly, 2 μg of each plasmid and 5 μl Lipofectamin 3000 were diluted in 125 μl Opti-MEM (Invitrogen) and the mixture of plasmid and reagent was incubated for 20 min at room temperature. Then the DNA-lipid complex was added dropwise into each well of cells along with gentle mixing after which the cells were incubated at optimal conditions. At 24 h post transfection, the culture medium was removed then the cells were further cultured until harvested.

### Co-immunoprecipitation (Co-IP) assays

HEK293T/17 cells were grown in 100 mm tissue culture plates and mock transfected or transfected pCDH-EF1-FHC_PHB1 in parallel with the empty plasmid vector as a negative control for 24 h, prior to being mock infected or infected with DENV 2, or DENV 4 according to the standard protocol. On day 3 post-infection, cells were collected and lysed with lysis buffer (50 mM Tris–HCl pH 7.5, 150 mM NaCl, 1% NP-40, 0.5 mM EDTA, 0.5 mM activated Na_3_VO_4,_ PIC). A total of 500 μl of lysate was pre-cleared by incubation with Protein G Sepharose 4 Fast Flow media (GE Healthcare, Buckinghamshire, UK) at 4 °C and with rotation for 3 h. Consequently, 100 μl of pre-cleared lysates from infection or mock infection were incubated with or without the pulldown antibody as given in Supplemental Table S2 with gentle rocking at 4 °C overnight. Next, the mixture was incubated with 20 μl of a 50% slurry of protein G Sepharose beads with gentle rocking at 4 °C overnight. Subsequently, the mixture was centrifuged at 6000×*g* for 3 min at 4 °C and the supernatant was removed. Then the pellets were washed five times with lysis buffer, and resuspended with 3× SDS sample buffer. The samples were heated at 100 °C for 5 min followed by centrifugation at 14,000×*g* for 3 min at 4 °C, and finally the samples were analyzed by SDS-PAGE and transferred to solid matrix membranes before western blot assay.

### Immunofluorescence assay

HEK293T/17 cells were plated on cover slips in six-well tissue culture plates and incubated under standard conditions until 60% confluence was reached, after which cells were mock transfected or transfected and after 24 h subsequently mock infected or infected as described above. At 72 h post mock infection or infection, the cell culture media was removed and cells were washed with 1XPBS followed by fixing with 4% paraformaldehyde in 1XPBS at room temperature for 20 min. Subsequently, cells were washed twice with 1XPBS/IFA (0.05 M NaH_2_PO_4_, 0.05 M Na_2_HPO_4_, 0.154 M NaCl) for 5 min and permeabilized with 0.3% TritonX-100 in 1XPBS/IFA for 10 min followed by washing twice with 0.03% TritonX-100 in 1XPBS/IFA for 5 min. Cells were blocked with 5% BSA in 1XPBS/IFA for 30 min and washed twice with 1XPBS/IFA for 5 min. Next, cells were incubated with the appropriate primary antibodies (Supplementary Table S3) at 4 °C overnight. After washing, cells were incubated with the appropriate secondary fluorescence conjugated antibodies (Supplementary Table 3), and a 1:100 dilution of 4′6-diamidino-2-phenyllindole (DAPI) at room temperature for 1 h in the dark. Slides were examined under ZEISS LSM 800 confocal microscope running ZEN (blue edition) version 2.3 (Carl Zeiss Microscope GmbH, Jena, Germany).

## Results

### 2D-gel and ontological analysis of differentiating host protein in HEK293T/17 cells after DENV 2 and DENV 4 infection

Several of our previous studies have shown that different DENV strains infecting cells in culture at the same MOI can induce extremely different levels of infection^18,20^. While varying MOI can be used to equalize infection, this has its own inherent problems in analysis and interpretation. We therefore selected to use a cell line that does not represent a target cell of DENV, but is generally uniformly infectable by DENV strains, and additionally is suitable for cellular transfection, namely HEK293T/17. We therefore undertook an optimization of infection analysis (Supplemental Figs. S1 and S2), and results showed that HEK293T/17 cells infected with either DENV 2 or DENV 4 at MOI 5 produced infection levels that were not significantly different on day 3 post infection (Supplementary Fig. S3).

**Figure 1:**
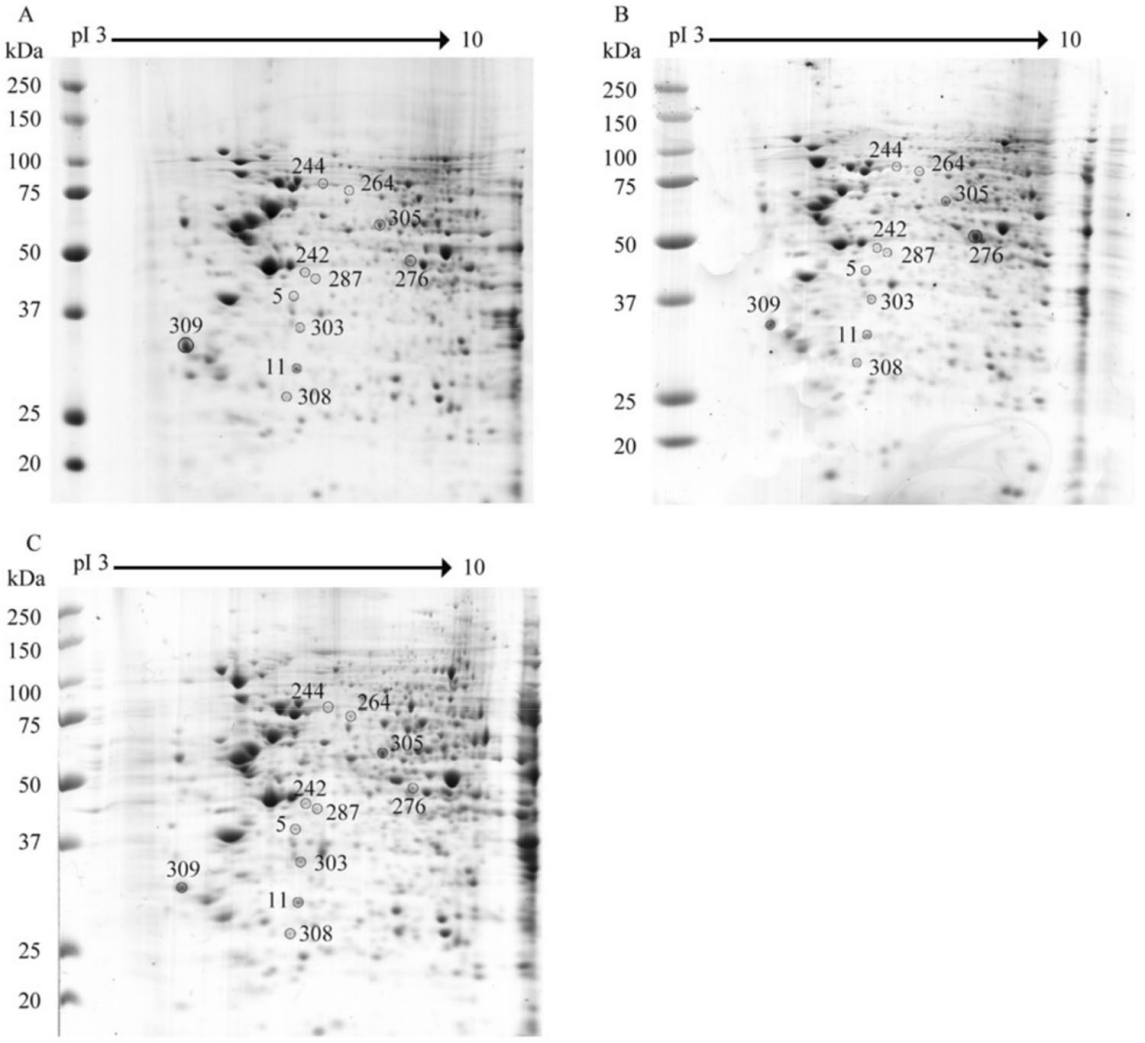
Representative two-dimensional polyacrylamide gels of HEK293T/17 cells after mock or DENV 2 or DENV 4 infection. HEK293T/17 cells were either mock infected (**A**), or infected with DENV 2 (**B**) or DENV 4 (**C**) and at 72 h post-infection cells were harvested, proteins prepared and 250 µg of proteins were subjected to 2D proteomic analysis. Gels were subsequently stained with Coomassie blue G250. Experiment was undertaken independently in triplicate. Intensity of protein spots were analyzed using ImageMaster 2D Platinum version 7.0 software. The spots intensity was determined by student’s t test with a *p* value of less than 0.05 were considered as statistically significant. All 2-D gels can be found in the supplemental materials (Supplemental Figs. S4–S6).

HEK293T/17 cells were therefore either mock infected or infected with DENV 2 or DENV 4 at MOI 5, independently in triplicate. The cells were collected at 72 h post infection after which proteins were extracted. Protein samples were subjected to 2D-gel electrophoresis followed by staining with Coomassie Blue G250 (Fig. 1). A total of 293 protein spots were seen in samples from mock infection, 287 protein spots were seen in samples after DENV 2 infection and 319 spots were seen in samples after DENV 4 infection. The intensity of the protein spots was analyzed using ImageMaster 2D Platinum version 7.0 software, and significance was determined by student’s t test with a p value of less than 0.05 being considered as statistically significant.

Analysis of the results showed that only 11 spots were significantly differentially regulated by both DENV 2 and DENV 4 infection (with each being compared to mock infection). The 11 significantly differentially expressed spots were cut and subjected to identification by mass spectrometry, and all protein spots were successfully identified (Table 1). Six of the proteins were consistently differentially regulated with the protein either being up regulated by infection with both DENVs (four proteins namely 26S proteasome non-ATPase regulatory subunit 13, Complement component 1 Q subcomponent-binding protein, T-complex protein 1 subunit beta, Eukaryotic translation initiation factor 3 subunit I) or down regulated by infection with both DENVs (two proteins namely Prohibitin and Ubiquitin carboxyl-terminal hydrolase isozyme L1). The remaining five identified proteins (Pyruvate dehydrogenase E1 component subunit alpha, Leukotriene A-4 hydrolase, Inorganic pyrophosphatase, COP9 signalosome complex subunit 4 and Heat shock 70 kDa protein 1A) showed discordant differential expression with the protein being significantly up-regulated by one DENV, and significantly down-regulated by the other DENV (Table 1).

**Table 1:** Identified proteins which are differentially expressed after DENV 2 or DENV 4 infection in HEK293T/17 cells.

No.	spot ID	Protein name	pI	Mass	Intensity					*P* value
					mock	DENV-2	DENV-4	Fold change		
								DENV-2/mock	DENV-4/mock	
1	276	Pyruvate dehydrogenase E1 component subunit alpha	8.35	43,952	2036.28	593.12	3490.80	0.291	1.714	6.04E−04
2	287	26S proteasome non-ATPase regulatory subunit 13	5.53	43,203	654.04	857.56	2102.10	1.311	3.214	0.001246
3	264	Leukotriene A-4 hydrolase	5.8	69,868	621.78	1470.95	205.50	2.366	0.330	0.001748
4	303	Inorganic pyrophosphatase	5.54	33,095	953.55	815.51	2118.92	0.855	2.222	0.005726
5	11	Prohibitin	5.57	29,844	3414.86	1933.45	1824.49	0.566	0.534	0.027197
6	309	Complement component 1 Q subcomponent-binding protein	4.74	31,749	414.11	3511.99	1072.91	8.481	2.591	0.027321
7	305	T-complex protein 1 subunit beta	6.01	57,794	2278.44	2406.61	2514.45	1.056	1.104	0.039927
8	242	COP9 signalosome complex subunit 4	5.57	46,525	1812.78	607.079	2412.74	0.335	1.331	0.043057
9	244	Heat shock 70 kDa protein 1A	5.48	70,294	1925.58	1435.79	2615.88	0.746	1.358	0.045142
10	5	Eukaryotic translation initiation factor 3 subunit I	5.38	36,878	580.443	1288.74	2632.36	2.220	4.535	0.049521
11	308	Ubiquitin carboxyl-terminal hydrolase isozyme L1	5.33	25,151	2508.73	2255.80	1416.10	0.899	0.564	0.049756

**Figure 2:**
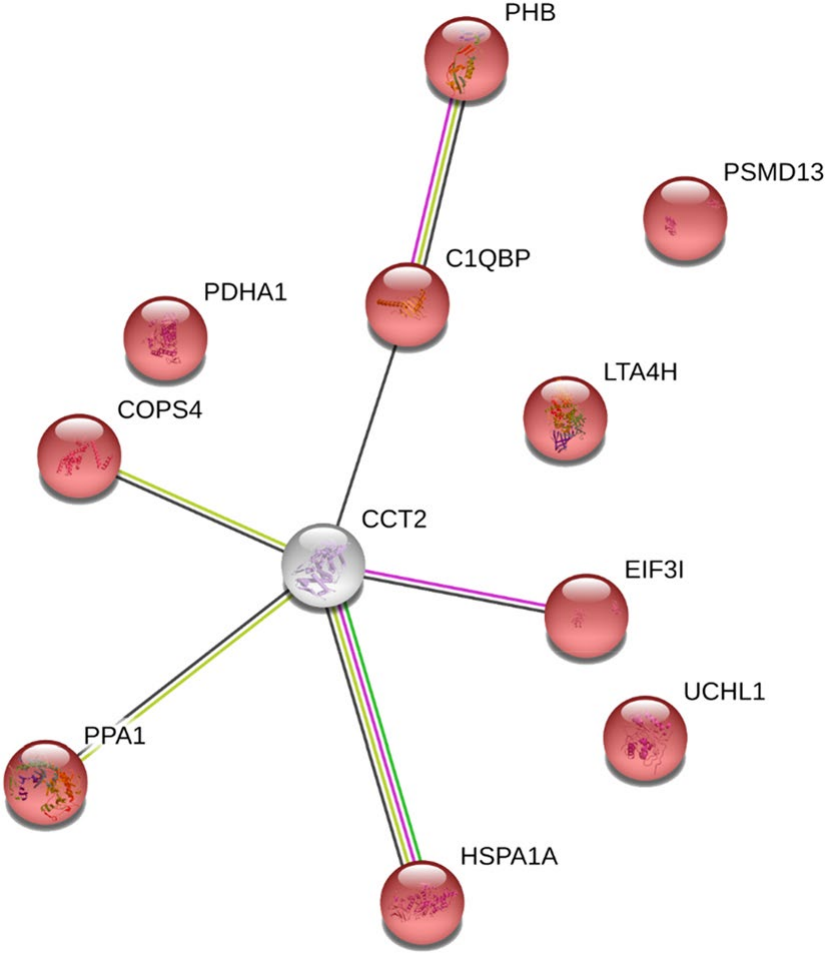
STRING analysis of significantly expressed protein after DENV 2 and DENV 4 infection. The 11 identified proteins from the 2D proteomic analysis were submitted to STRING database version 11.0, the network data shown highly significantly interaction. PPI enrichment p-value is 0.0109. The mainly functional enrichment is cellular metabolic process which are represented in red.

Overall, a STRING analysis (Fig. 2) revealed that the differentially expressed proteins after both DENV 2 and DENV 4 infection were mainly involved in cellular metabolism pathways (Supplemental Table S4). Additionally, a functional annotation clustering result from the DAVID bioinformatics resource confirmed the presence of mitochondrial proteins, and that the overall results were consistent with host-virus interactions in the biological process database (Supplemental Table S5).

### Validation of differential protein expression in HEK293T/17 cells after DENV 2 and DENV 4 infection

From the 2-D analysis, two candidate proteins, Hsp70 and prohibitin, were of particular interest as both have previously been implicated as playing roles in both DENV entry and interacting with DENV E protein. For example, Reyes-Del Valle and colleagues^25^ identified Hsp70 as part of a receptor complex mediating DENV entry to monocytes, although this complex did not seem to mediate DENV entry to hepatoma cells^26^. Similarly, prohibitin has been identified as a DENV receptor protein and interacting protein in insect cells^27^. In addition, inorganic pyrophosphatase (PPI), Hsp70 and the possible pathway proteins Hsp90 and stress induced phosphoprotein 1 (a protein that interacts with both Hsp70 and Hsp90 (reviewed in^28^) were evaluated for their expression.

**Figure 3:**
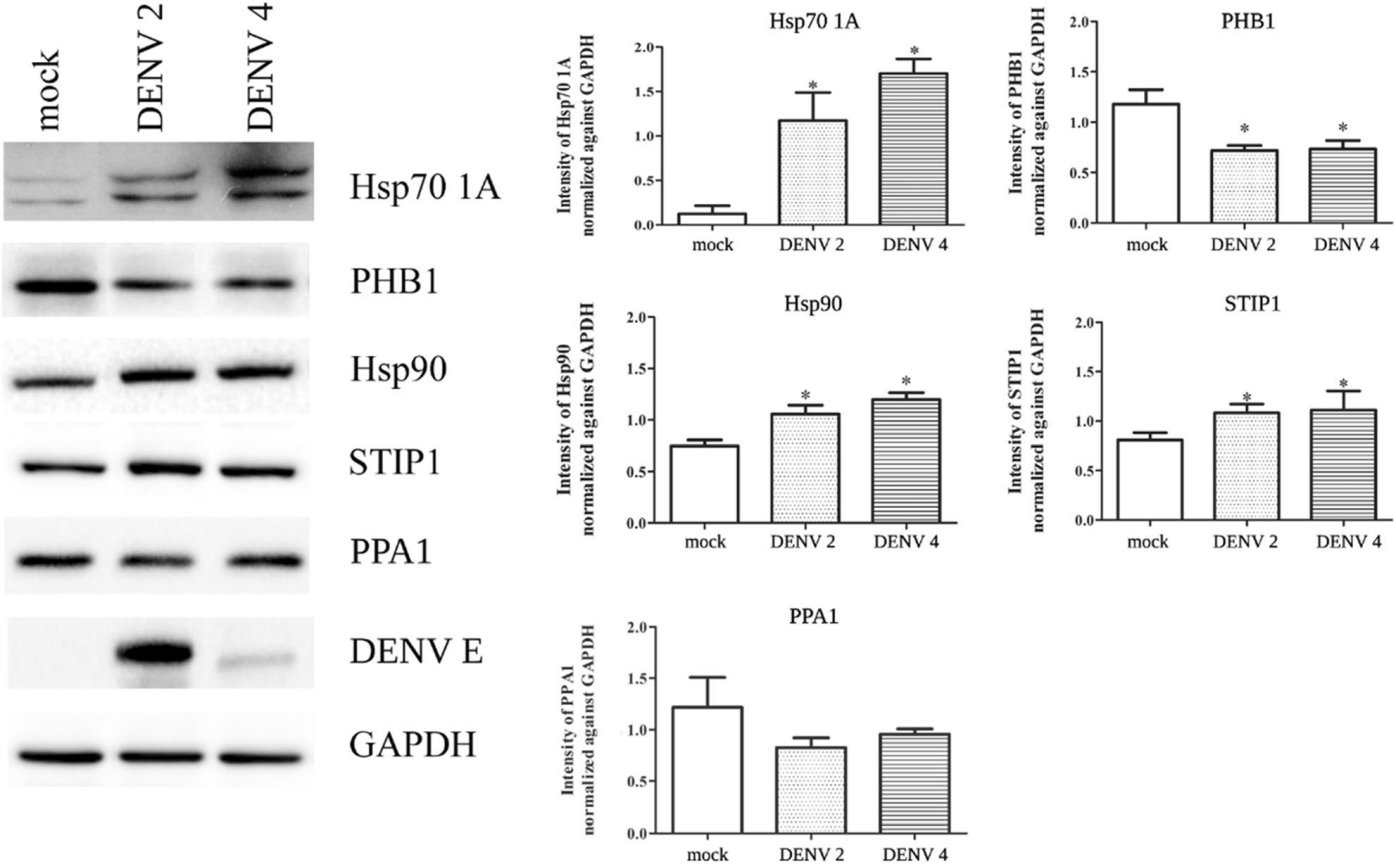
Validation of candidate proteins from 2D proteomic analysis. HEK293T/17 cells were either mock-infected or infected with DENV 2 or DENV 4 at MOI 5 for 72 h. Western blot analysis was performed to validate the expression of Hsp701A, PHB1, Hsp90, STIP1, PPA1 and STIP1. In addition gels were probed for DENV E protein and GAPDH as in internal control. All experiments were performed independently in triplicate. Protein band intensities were quantified using GraphPad Prism5 program and the expression of all proteins was normalized to GAPDH. Error bars represent S.E.M. *p* value * < 0.05. Full, uncropped western blots can be found in the supplemental materials.

HEK293T/17 cells were therefore either mock infected, or infected with DENV 2 or DENV 4 and at 72 h post infection cells were harvested, proteins were extracted and used in western blot analyses. The chaperone proteins, heat shock protein 70 kDa 1A (HSPA1A), heat shock protein 90 (HSP90), and stress induced protein1 (STIP1), were all significantly up-regulated in expression by both DENVs (Fig. 3). For Hsp70, this is discordant with the proteome analysis which showed significant down-regulation by DENV 2, and significant upregulation by DENV 4 infection (Table 1). Meanwhile, prohibitin1 (PHB1) was significantly down-regulated in expression by both DENV 2 and DENV 4 infection (Fig. 3), consistent with the results of the proteomic analysis (Table 1). Inorganic pyrophosphatase1 (PPA1) showed no significant change in expression after infection by either of the two DENVs (Fig. 3).

### Investigation of role of PHB1 in DENV infection

To investigate the role of PHB1 in DENV infection, PHB1 protein was over-expressed in HEK293T/17 cells before DENV infection. To achieve PHB1 over-expression HEK293T/17 cells were transfected with PHB1-pCDH-EF1-FHC in parallel with cells transfected with pCDH-EF1-FHC (empty vector control) and cells mock-transfected with Lipofectamine alone. At 24 h post transfection overexpression of PHB was confirmed by both western blotting and immunohistochemistry (Supplemental Fig. S7).

**Figure 4:**
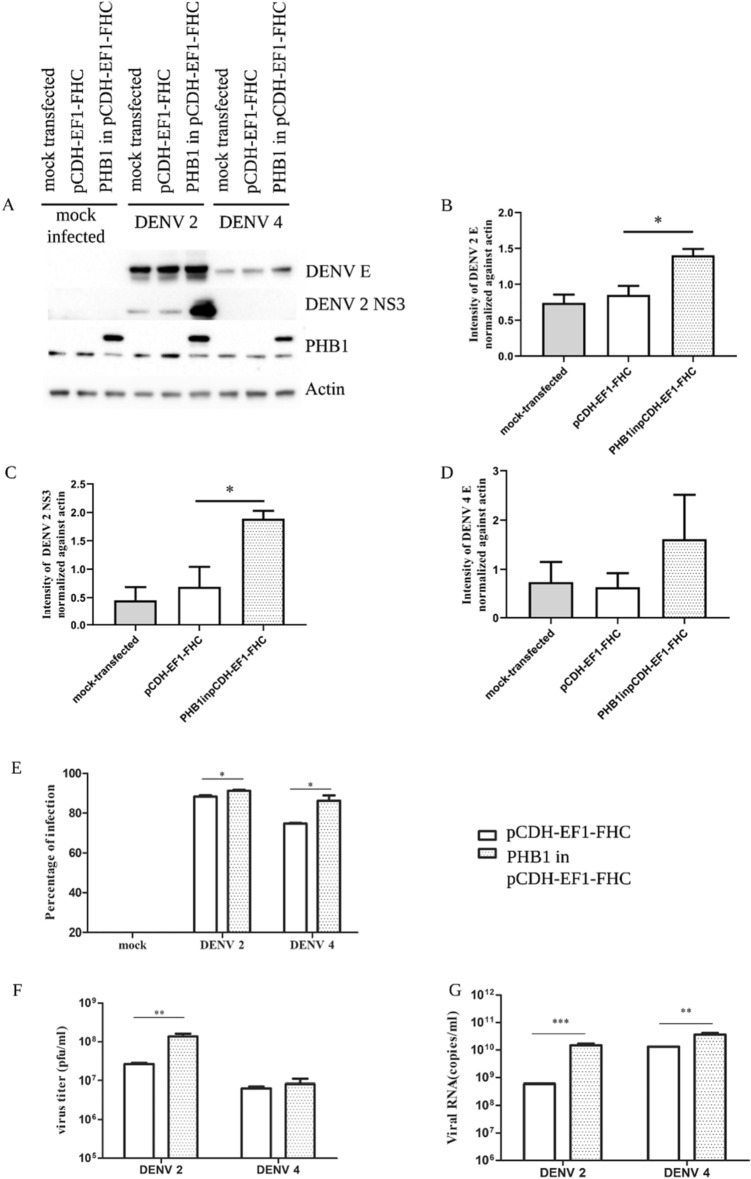
Determination the effects of PHB1 on DENV infection in HEK293T/17 cells. HEK293T/17 cells were mock transfected or transfected with 2 µg of a plasmid expressing PHB1 or an empty vector. At 24 h post-transfection cells were either mock-infected or infected with DENV 2 or DENV 4 at MOI 5 for 72 h. Subsequently proteins were extracted and used in (**A**) western blot analysis to detect expression of DENV E protein, DENV NS3 protein, PHB1 and actin. Protein bands were quantitated for (**B**) DENV 2 E protein, (**C**) DENV 2 NS3 protein and (**D**) DENV 4 E protein. In parallel (**E**) percentage of infection (**F**) virus titer and (**G**) cellular viral genome copy number were determined. All experiments were undertaken independently in triplicate. Error bars represent S.E.M. *p* value ** < 0.01 and * < 0.05. Full, uncropped western blots can be found in the supplemental materials.

At 24 h post-transfection, cells were either mock infected, or infected with DENV 2 or DENV 4 at MOI5. On day 3 post-infection, cells were harvested, proteins were prepared and separated by electrophoresis through 12% polyacrylamide gels and then transferred to a solid matrix. The membranes were probed with antibodies against human PHB1, dengue E protein, DENV 2 NS3 protein and actin as an internal control. Results (Fig. 4A–D) showed that DENV E protein of both DENVs was increased in PHB transfected cells. The increased viral protein expression was most clearly seen with DENV 2 NS3, albeit that the antibody directed against DENV 2 NS3 did not cross react with DENV 4 NS3 protein. Consistently, cells over expressing PHB1 showed significantly increased viral infectivity and viral RNA copy number in both DENV 2 and DENV 4 infection (Fig. 4E,G). Notably, a significant increase in viral production in PHB1 transfected cells as compared to empty vector transfected cells was seen with DENV 2, but not with DENV 4 (Fig. 4F). Consistently however, the results are consistent with PHB1 having a role in DENV infection.

### Co-localization and intracellular interaction between PHB1 and E protein of DENV 2 and DENV 4

**Figure 5:**
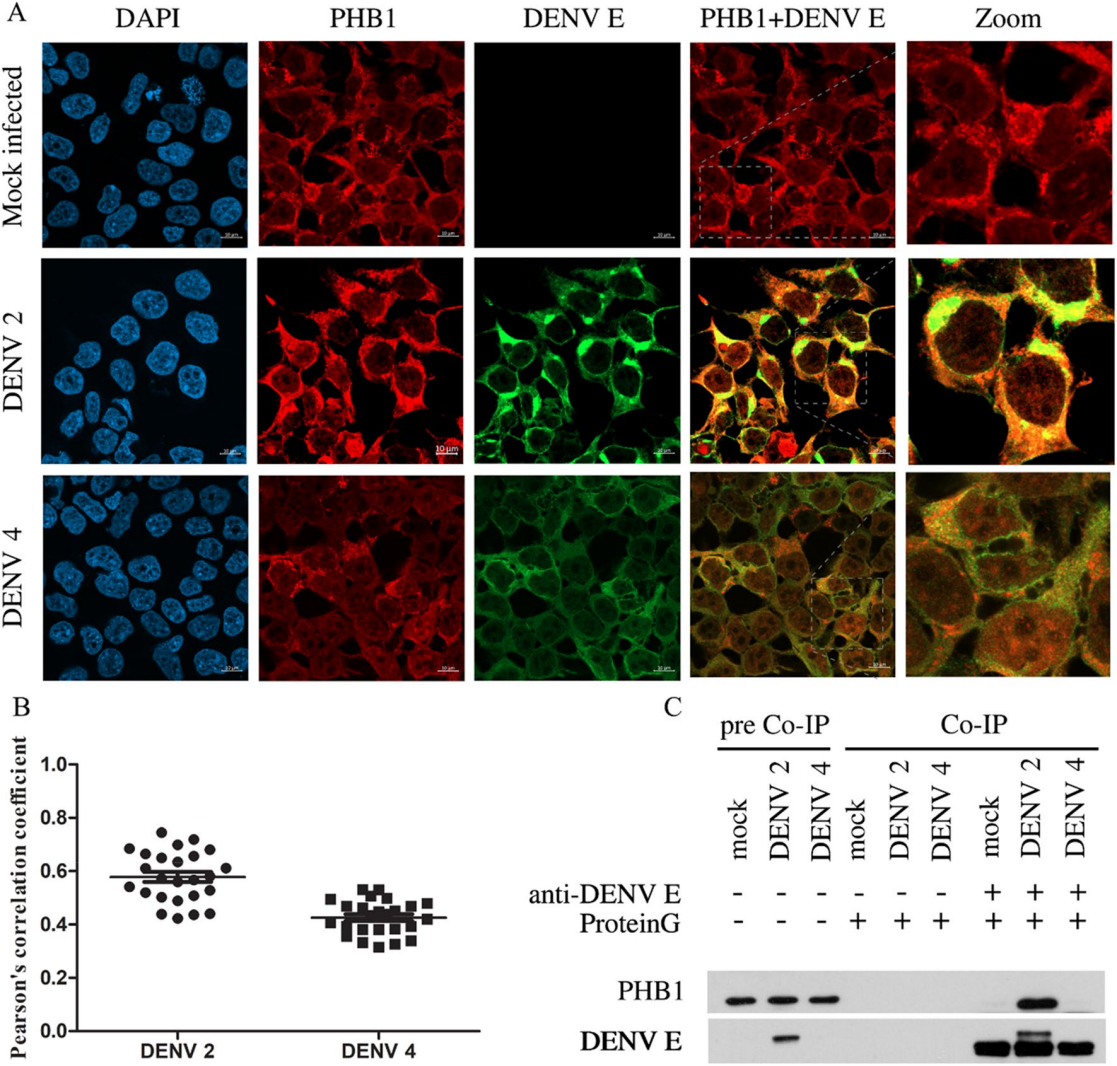
Co-localization and interaction of PHB and DENV E protein. (**A**) HEK293T/17 cells were grown on cover slips and then either mock infected or infected with DENV 2 or DENV 4 at MOI 5. At 72 h post infection, the cells were fixed, permeabilized and incubated with a polyclonal anti-PHB1 antibody (RED) and pan specific anti-dengue E protein (GREEN) follow by appropriate fluorescent conjugated secondary antibodies followed by staining with DAPI (BLUE). Cells were examined under a ZEISS LSM 800 confocal microscope co-localization between PHB1 and DENV E protein shows in yellow. (**B**) The degree of colocalization between PHB1 and DENV E protein was assessed by determining the Pearson’s correlation coefficient. (**C**) The interaction between DENV E protein and PHB1 was investigated by immunoprecipitation. HEK293T/17 cells were mock infected or infected with DENV 2 or DENV 4 and at 72 h post infection protein lysates were prepared. DENV E protein was pulled down with an anti-dengue E protein antibody, and the presence of co-immunoprecipitated PHB1 determined by western blot analysis. Full, uncropped western blots can be found in the supplemental materials.

To determine any colocalization between PHB1 and DENV E protein, HEK293T/17 cells were seeded on cover slips and transfected with PHB1 constructs for 24 h, then transfected cells were infected with DENV 2 or DENV 4 in parallel with mock-infection. At 72 h post infection, cells were fixed with 4% paraformaldehyde and permeabilized with 0.3% Triton X-100. Subsequently, cells were stained with an anti-human PHB1 antibody, an anti-HA antibody, and an anti-DENV E protein antibody, followed by an appropriate secondary antibody conjugated with fluorescence dyes (Supplemental Table S3). Cells were finally counter stained with DAPI. The results showed significant co-localization between PHB1 and both DENV 2 and DENV 4 E protein (Fig. 5A) with Pearson’s correlation coefficients of 0.6 and 0.4, respectively (Fig. 5B).

To determine whether there is a direct intracellular interaction between PHB1 and DENV E protein, coimmunoprecipitation experiments were performed. HEK293T/17 cells were infected with either mock infected or infected with DENV 2 and DENV 4. On day 3 post infection, cells were harvested and lysed before incubation with a sepharose slurry of protein G followed by incubating with a goat polyclonal anti-PHB1 antibody, followed by a second round of incubation with protein G sepharose slurry. Subsequently, coimmunoprecipitated proteins were run on polyacrylamide gels prior to transfer into solid matrix then DENV E and PHB1 protein signals were detected. The result showed that only DENV 2 E protein was coimmunoprecipitated with PHB1 protein (Fig. 5C).

## Discussion

The viral species *Dengue virus* is composed of four different viruses (DENV 1 to DENV 4), and each of the DENVs is further composed of multiple distinct lineages, which are themselves composed of many different strains. Upon infection with one DENV, a robust, long term adaptive immunity is generated, which prevents further infection with that particular DENV. However, there is no (or possibly only very limited duration) protection against other, heterotypic DENVs^29^. The initial adaptive immune response generates antibodies that can cross-react with other DENVs, but these cross-reactive antibodies do not neutralize the second infection. Instead they can potentiate the infection by allowing entry of the virus to Fc receptor bearing cells, in a process known as antibody dependent enhancement of infection^30^. This process has significantly complicated vaccine design as a vaccine must be able to induce a robust antibody response to all four DENVs simultaneously. This has proved problematic in practice^31,32^. In parallel, despite years of development no specific drug has been developed to treat DENV infection. The influence of variation (DENV, lineage, strain) on potential drug treatments remains largely unexplored, but in a recent study we showed that different DENVs showed markedly different responses to the anti-obesity drug orlistat^20^. In attempting to understand the mechanism of DENV infection, and to possibly identify novel druggable targets, a number of studies have investigated proteomic changes in DENV infected cells in vitro^9–14^. Markedly, all of the studies have utilized DENV 2 as the infecting virus, and as such any differences based on infecting virus remain unknown. In a previous study, we showed that an LC–MS/MS analysis on pooled sub-10 kDa fractions from 12 different DENVs representing all four DENVs and multiple strains for each DENV identified 128 proteins, of which only 20% were consistently expressed in all samples, while the remaining 80% were variably expressed^18^. That study suggested that common druggable targets may be difficult to find. To explore this further, we undertook a direct comparative 2D-proteomic analysis of HEK293T/17 cells infected with either DENV 2 or DENV 4. In each case, infected cells were compared to mock infected cells. Surprisingly, out of several hundred spots identified, only 11 were identified as being differentially regulated by both DENVs. Even more concerning was the fact that only 6 of the spots showed co-ordinate regulation (up or down in expression in response to both DENV infections). This further reinforces our prior observation that DENV/strain variation has a significant impact on the nature of the proteomic changes, and that “common” changes are the exception rather than the rule.

It has to be noted that the two viruses do have some differences beside serotype. The DENV 2 used here (strain 16681) is a laboratory adapted virus that has been continually cultured since 1984, while the DENV 4 used is a low passage isolate. We obtained both of these viruses with no supporting passage history, but it is sufficient to note that one is very high passage and one is low passage. While it is possible that this may affect the results, our earlier study using proteomic analysis of 12 DENV of different passage history showed no clustering of the viruses in a PCA analysis according to serotype, or, importantly passage history^18^.

Protein validation showed that the chaperone proteins HSPA1A, HSP90b and STIP1 were all significantly up-regulated in expression by both DENVs. For Hsp70, this was discordant with the proteome analysis which showed significant down-regulation by DENV 2, and significant upregulation by DENV 4 infection. However, the role of chaperone proteins in DENV infection has been well documented, and it is perhaps unsurprising that this group of proteins is found as one of the few consistent results when comparing between DENV 2 and DENV 4.

Of the four proteins showing contrasting changes in expression (after Hsp70 showed co-ordinate regulation in the western blot validation) in the proteome analysis (pyruvate dehydrogenase E1 component subunit alpha, leukotriene A-4 hydrolase, inorganic pyrophosphatase and the COP9 signalosome complex subunit 4) it is difficult to know why the expression is contrasting. It is possible that like HSPA1A a fuller evaluation would show that they are indeed consistent, although the current results fit with our previous study^18^. A David functional annotation clustering ontology analysis (Supplemental Tables S4 and S5) indicated that the proteins were collectively associated with the process of cellular and nuclear acetylation, and acetylation of the NS3 helicase domain is known to be essential for flavivirus replication^33^, but again how discordant expression of these proteins affect viral replication remains unclear.

PHB1 was significantly down-regulated in expression by both DENV 2 and DENV 4 infection consistent with the results of the proteomic analysis. Prohibitin consists of two proteins, PHB1 and PHB2, which are homologous to each other, but have slightly different molecular weights, with PHB1 having a mass of about 30 kDa, while the mass of PHB2 is about 37 kDa^34^. The two proteins form oligomers and hetero-oligomerization has been suggested to be required for stability of the protein, as loss of PHB1 leads to loss of PHB2, and vice versa^35–39^. PHB is found in several cell compartments, particularly the mitochondria^40–42^, but is also present in the cytoplasm and nucleus^43,44^ as well as at the plasma membrane^45^. PHB1 was initially identified as a negative regulator of proliferation^46^. Since then, PHB has been proposed to be involved in a diverse range of biological processes including mitochondrial function, cell proliferation and development as well having a role in PI3K-Akt-mTOR signaling through an interaction with FKBP8^47^ and in the Raf/ERK signaling pathway^48^.

There have been few reports of the involvement of PHB in DENV infection. One report identified PHB as a DENV receptor protein expressed on the surface of insect cells^27^, and other studies have supported the possible involvement of PHB in infection of insect cells by identifying PHB as a DENV E protein interacting protein expressed on the cell surface^49^. One further study has identified prohibitin as a receptor molecule mediating entry of DENV 3 into human neuroblastoma (SH-SY5Y) and microglial (CHME-3) cells^50^.

In this study PHB was identified as one of only 6 proteins (26S proteasome non-ATPase regulatory subunit 13, Complement component 1 Q subcomponent-binding protein, T-complex protein 1 subunit beta, Eukaryotic translation initiation factor 3 subunit I Prohibitin1 and Ubiquitin carboxyl-terminal hydrolase isozyme L1) that showed coordinate regulation by both DENV 2 and DENV 4. The 2D analysis identified this protein as down-regulated in infection, and this was confirmed in a validating western blot analysis. Markedly, over-expression of PHB1 resulted in a significant increase in the level of infection and in viral genome copy number for both DENV 2 and DENV 4. Infectious titer was significantly increased in DENV 2 infection, and increased slightly (but not significantly) in DENV 4 infection. These results suggest that down-regulation of PHB1 is a cellular antiviral mechanism.

Both DENV 2 ad DENV 4 E proteins co-localized with prohibitin, albeit that greater co-localization was seen with DENV 2 E protein as compared to DENV 4 E protein. One possible reason for this was that there was a clear interaction between DENV 2 E protein and PHB, while we could not confirm the interaction with DENV 4 E protein. Markedly, the interaction between DENV 2 E protein and PHB is consistent with previous studies^27^.

## Conclusion

The species *Dengue virus* presents unique challenges in developing both effective vaccines and therapeutics. This is a consequence of divergent viruses (DENV 1 to 4), multiple lineages and numerous identified strains. As with other positive sense RNA viruses the DENV RNA dependent RNA polymerase (part of NS5) lacks any proof reading function, leading to error prone replication^51^ that can serve to drive further diversity. However, how this diversity affects cellular interactions remains largely unknown. Utilizing a 2D proteomic analysis of DENV 2 and DENV 4 infection only 6 coordinately regulated proteins were detected. This represents only some 1.5% of the protein spots identified. Even then, when PHB was identified as a coordinately regulated protein that was down regulated by both DENV 2 and DENV 4 infection and was shown to colocalize with both E proteins a direct interaction between DENV E protein and PHB was only seen for DENV 2 E protein and not DENV 4 E protein. These results further confirm our proposal that there are only a few consistent changes in DENV infection, and that the interaction is defined by one of “plasticity”^18^ in which the same effect can be achieved by different DENVs through divergent mechanisms.

### Supplementary Information

Supplementary Information.

## Data Availability

All data generated or analyzed during this study are included in this published article (and its Supplementary Information file).
