# Supplementary material for: A 2D-proteomic analysis identifies proteins differentially regulated by two different dengue virus serotypes
Source: Sci Rep. 2024 Apr 9;14:8287. doi: 10.1038/s41598-024-57930-1 (PMC11003990; doi:10.1038/s41598-024-57930-1)

Supplemental Files

A 2D-proteomic analysis identifies proteins differentially regulated by two different dengue virus serotypes.

Chanida Chumchanchira[1], Suwipa Ramphan[2], Atchara Paemaee[3], Sittiruk Roytrakul[3], Pathrapol Lithanatudom[4*], Duncan R. Smith[2*]

[1]PhD Degree Program in Biology, Faculty of Science, Chiang Mai University, Chiang Mai, 50200, Thailand

[2]Institute of Molecular Biosciences, Mahidol University, Nakhon Pathom, 73170, Thailand.

[3]National Center for Genetic Engineering and Biotechnology (BIOTEC), National Science and Technology Development Agency, Pathum Thani, 12120, Thailand

[4]Department of Biology, Faculty of Science, Chiang Mai University, Chiang Mai, 50200, Thailand.

***Correspondence to:** Pathrapol Lithanatudom (pathrapol_li@hotmail.com) or Duncan R. Smith (duncan_r_smith@hotmail.com).

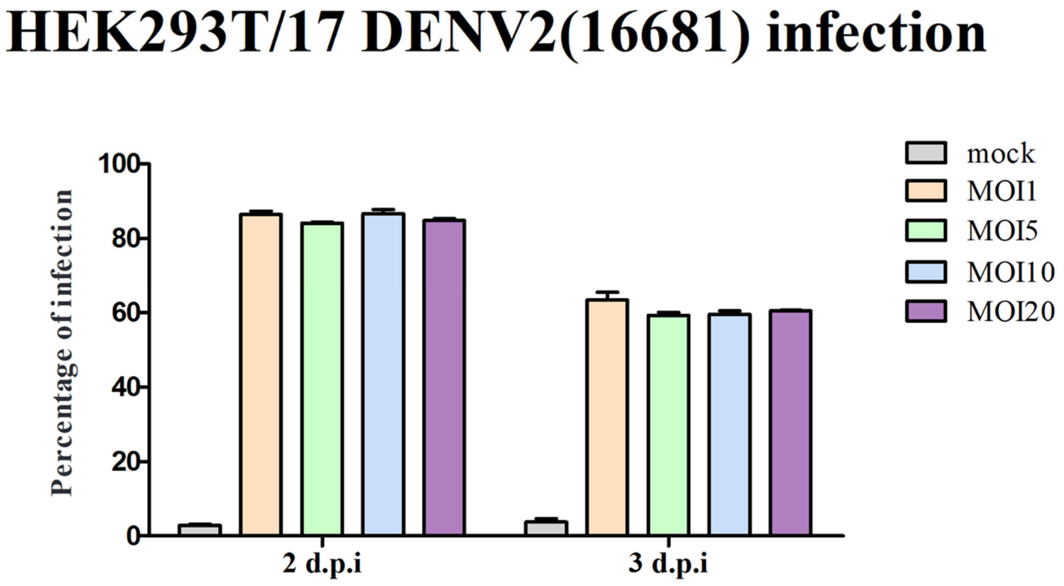

Supplemental Figure S1. Optimization of DENV 2 infection of HEK293T/17 cells.

HEK293T/17 cells were mock infected or infected with DENV 2 at different MOIs and the level of infection determined by flow cytometry at 48 and 72 hours post infection. Experiments were undertaken independently in triplicate.

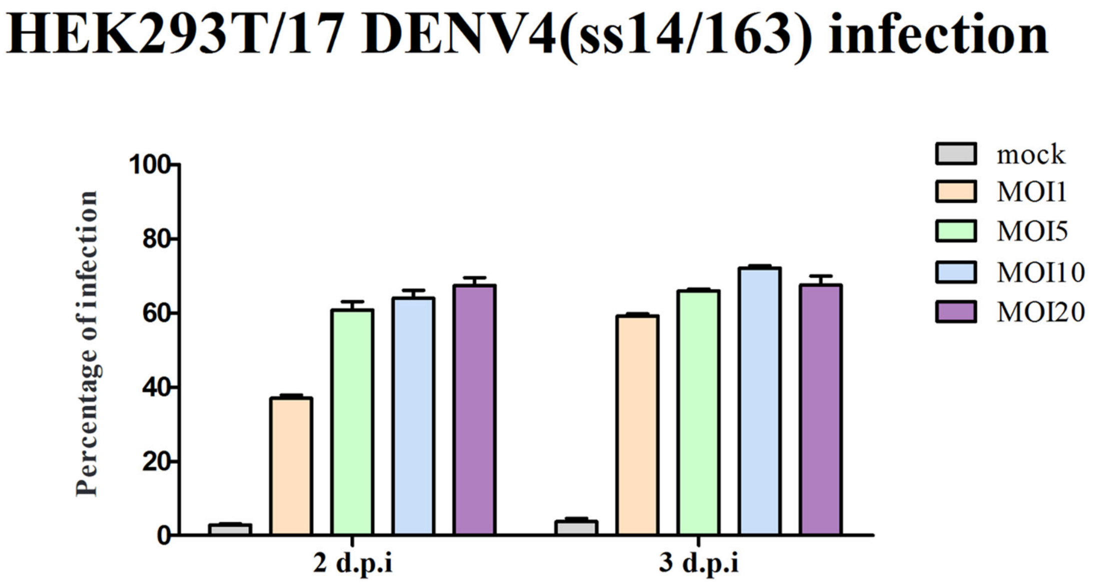

Supplemental Figure S2. Optimization of DENV 4 infection of HEK293T/17 cells.

HEK293T/17 cells were mock infected or infected with DENV 2 at different MOIs and the level of infection determined by flow cytometry at 48 and 72 hours post infection. Experiments were undertaken independently in triplicate.

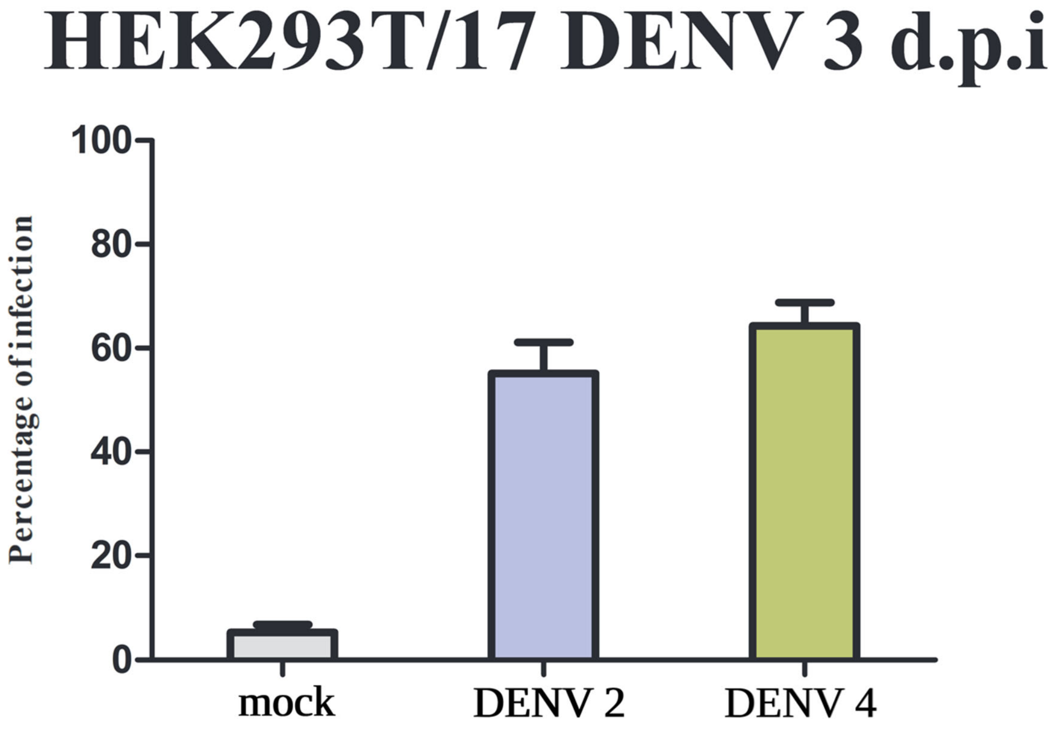

Supplemental Figure S3. Optimization of DENV 2 and 4 infection of HEK293T/17 cells.

HEK293T/17 cells were mock infected or infected with DENV 2 or DENV 4 at MOI 5 and the level of infection determined by flow cytometry at 72 hours post infection. Experiments were undertaken independently in triplicate.

Replicate 1

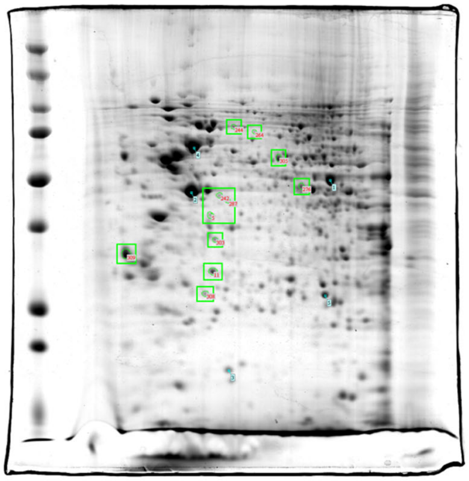

Replicate 2

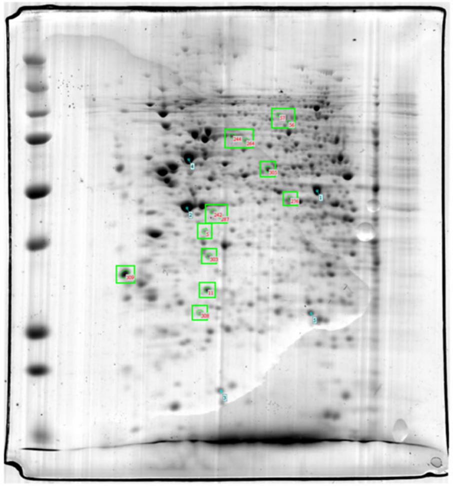

Replicate 3

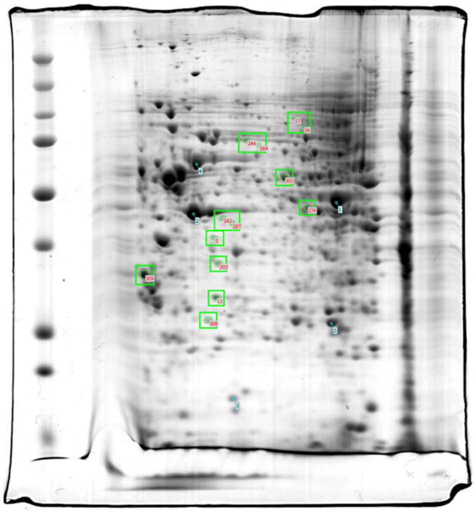

Supplemental Figure S4. Replicate 2D gels.
Three replicate 2D gels of HEK293T/17 cells mock infected at 72 hours post mock infection.

Replicate 1

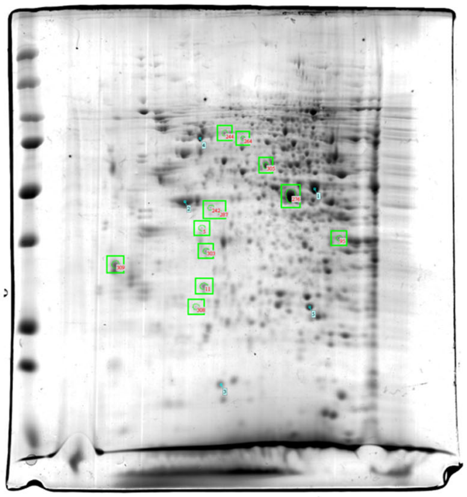

Replicate 2

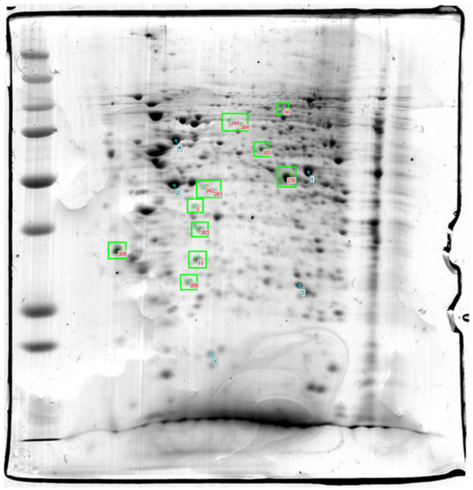

Replicate 3

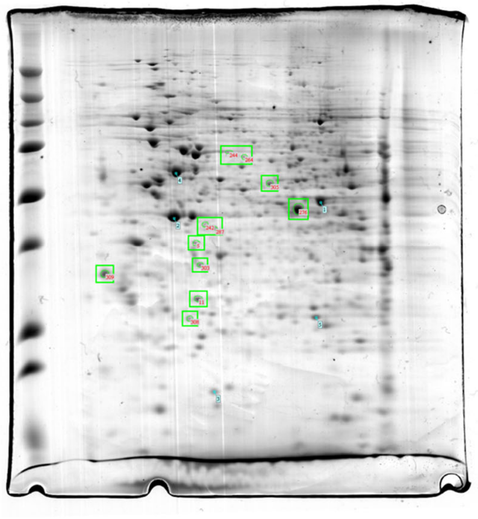

Supplemental Figure S5. Replicate 2D gels.
Three replicate 2D gels of HEK293T/17 cells infected with DENV 2 at 72 hours post infection.

Replicate 1

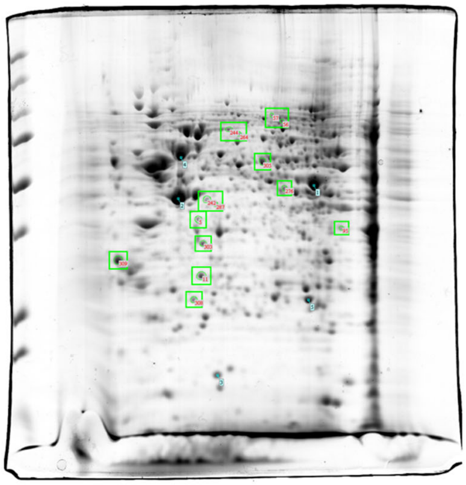

Replicate 2

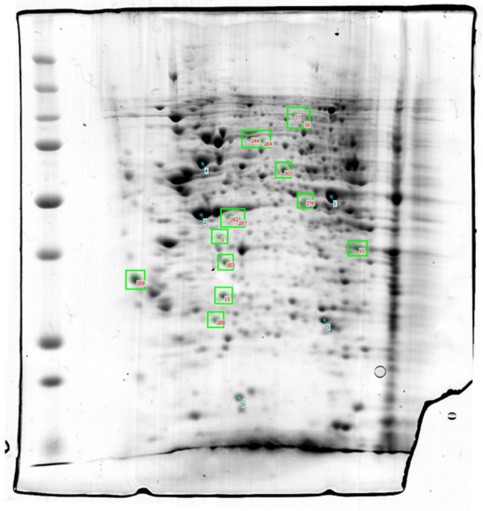

Replicate 3

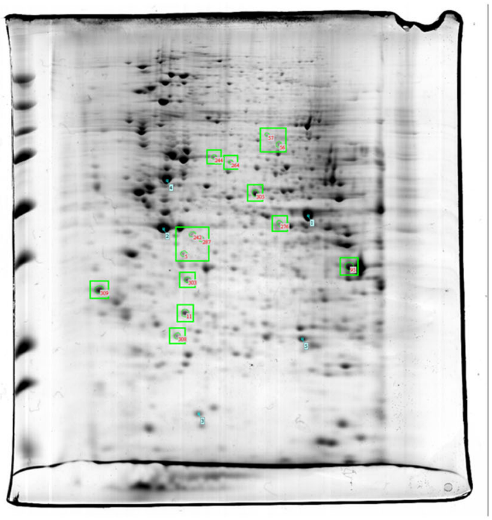

Supplemental Figure S6. Replicate 2D gels.
Three replicate 2D gels of HEK293T/17 cells infected with DENV 4 at 72 hours post infection.

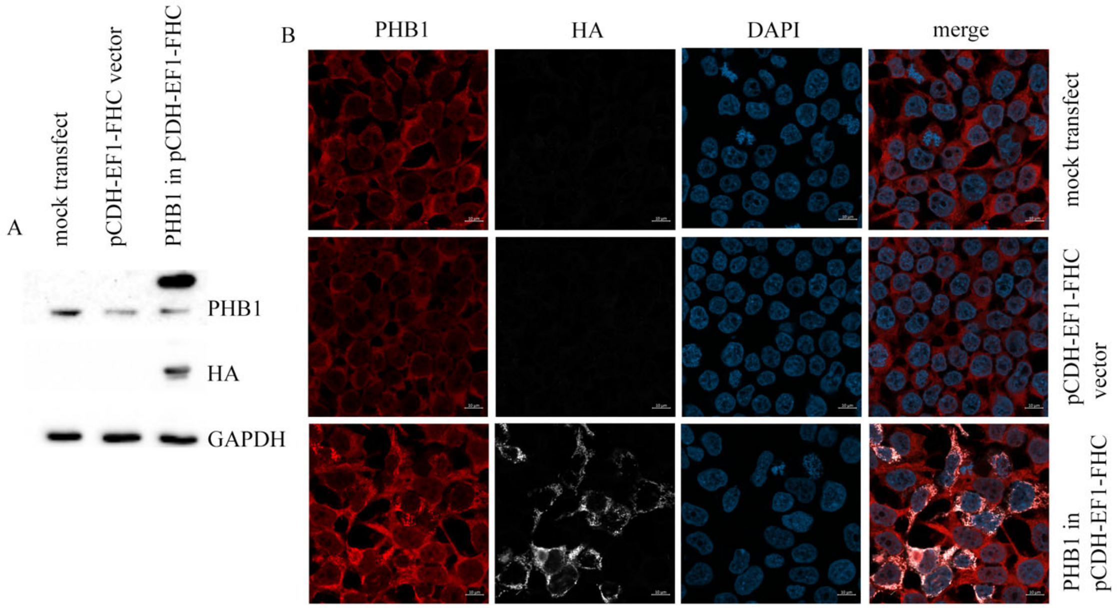

Supplemental Figure S7. Over-expression of PHB1.

HEK293T/17 cells were transfected with PHB1-pCDH-EF1-FHC in parallel with cells transfected with pCDH-EF1-FHC (empty vector control) and cells mock-transfected with Lipofectamine alone. At 24 hours post transfection overexpression of PHB was confirmed by both (A) western blotting and (B) immunohistochemistry.

Supplemental Table S1. Antibodies used in western blots

Name of antibody	Source	Type	Dilution	Detail
Anti-HSPA1A (PA5-34772; Pierce, Rockford, IL, USA)	rabbit	polyclonal	1:3000	1°Ab
Anti-Hsp90α/β (H-114): (sc-7947) Santa Cruz, Biotechnology Inc., Texas, USA	rabbit	polyclonal	1:10000	1°Ab
Anti-STIP1 (ab56873; Abcam plc, Cambridge, UK)	mouse	polyclonal	1:5000	1°Ab
Anti-PHB1 (N-20): (sc-18196) Santa Cruz, Biotechnology Inc., Texas, USA	goat	polyclonal	1:500	1°Ab
Anti-PPA1 (ab96099; Abcam plc, Cambridge, UK)	rabbit	polyclonal	1:3000	1°Ab
Anti-DENV 2 NS3 (PA5-32199; Pierce, Rockford IL)	rabbit	polyclonal	1:10,000	1°Ab
Anti-GAPDH (6C5): (sc-32233) Santa Cruz, Biotechnology Inc., Texas, USA	mouse	polyclonal	1:8000	1°Ab
Pan-specific anti-flavivirus E protein monoclonal antibody produced in house from hybridoma HB112	mouse	polyclonal	1:500	1°Ab
HRP-conjugated goat anti-mouse IgG (A4416; Sigma, MO, USA)	goat	polyclonal	1:5000	2°Ab
HRP-conjugated goat anti-rabbit IgG (31460; Pierce, IL, USA)	goat	polyclonal	1:5000	2°Ab
HRP-conjugated rabbit anti-goat IgG (31402; Pierce, IL, USA)	rabbit	polyclonal	1:5000	2°Ab

Supplemental Table S2. Antibodies used in co-immunoprecipitation assay

Name of antibody	Source	Type	Dilution/ concentration	Detail
Anti-PHB1 (N-20): (sc-18196) Santa Cruz, Biotechnology Inc., Texas, USA	goat	polyclonal	1ug	pull down
Pan specific anti-dengue virus type 1-4 antibody (MA1-27093; Pierce, Rockford, IL, USA)	mouse	polyclonal	1:500	1°Ab
HRP-conjugated goat anti-mouse IgG (A4416; Sigma, MO, USA)	goat	polyclonal	1:5000	2°Ab
HRP-conjugated goat anti-rabbit IgG (31460; Pierce, IL, USA)	goat	polyclonal	1:5000	2°Ab

Supplemental Table S3. Antibodies used in immunofluorescence assays

Name of antibody	Source	Type	Dilution	Detail
Anti-PHB1 (N-20): (sc-18196) Santa Cruz, Biotechnology Inc., Texas, USA	goat	polyclonal	1:50	1°Ab
Pan specific anti-dengue virus type 1-4 antibody (MA1-27093; Pierce, Rockford, IL, USA)	mouse	polyclonal	1:100	1°Ab
Anti-HA tag (ab9110 Abcam plc, Cambridge, UK)	rabbit	polyclonal	1:50	1°Ab
Alexa Fluor® 488 donkey anti-mouse IgG antibody (A11029, Invitrogen)	donkey	polyclonal	1:100	2°Ab
Alexa Fluor® 568 donkey anti-goat IgG antibody (A11057, Invitrogen)	donkey	polyclonal	1:100	2°Ab
Alexa Fluor® 647 donkey anti-rabbit IgG antibody (A31573, Invirtrogen)	donkey	polyclonal	1:100	2°Ab

Supplemental Table S4: Antibodies used in flow cytometry

Name of Antibody	Source	type	Dilution/concentration	Detail
Pan-specific mouse anti-dengue virus E protein monoclonal antibody produced by hybridoma HB114	mouse	monoclonal	1:150	1°Ab
FITC-conjugated goat anti-mouse IgG antibody (sc2010, Santa Cruz Biotechnology Inc)	Goat	polyclonal	1:40	2°Ab

Supplemental Table 5. Functional enrichments in biological processes analyzed by the STRING bioinformatic analysis software.

Biological Process(GO)			
Pathway ID	Pathway discription	Count*	FDR**
GO:1901564	organonitrogen compound metabolic process	10	0.0117
GO:0051131	chaperone-mediated protein complex assembly	2	0.0121
GO:0019538	protein metabolic process	8	0.0183
GO:0006955	immune response	5	0.0296
GO:0044267	cellular protein metabolic process	7	0.0328
GO:0044260	cellular macromolecule metabolic process	9	0.0328
GO:0044238	primary metabolic process	10	0.0453
GO:0044237	cellular metabolic process	10	0.0453

*Count: Count in gene set

**FDR: False discovery rate.

Supplemental Table 6. Functional annotation clustering results from the DAVID Bioinformatics Resource. Classification stringency: Medium.

**Annotation Cluster**	**Enrichment Score: 1.1**	**Count**	**P_value**
GOTERM_MF_DIRECT	transcription corepressor activity	3	4.70E-03
GOTERM_CC_DIRECT	mitochondria	4	2.90E-02
UP_KW_BIOLOGICAL_PROCESS	Host-virus interaction	3	4.50E-02
GOTERM_BP_DIRECT	negative regulation of transcription from RNA polymerase II promoter	3	8.90E-02
GOTERM_CC_DIRECT	cytoplasm	6	1.10E-01
GOTERM_CC_DIRECT	plasma membrane	4	5.00E-01
UP_KW_CELLULAR_COMPONENT	nucleus	4	6.80E-01

Supplemental Table S7. Functional annotation clustering results from the DAVID Bioinformatics Resource. Classification stringency: Medium.

Annotation Cluster 1	Enrichment Score: 1.09	Count	P_value
UP_KW_PTM	Acetylation	4	$1.5E^{-2}$
GOTERM_CC_DIRECT	cytosol	3	$1.8E^{-1}$
GOTERM_CC_DIRECT	cytoplasm	3	$1/9E^{-1}$

Supplemental Table S8. Functional annotation clustering results from the DAVID Bioinformatics Resource. Classification stringency: Medium.

Annotation Cluster 2	Enrichment Score: 0.88	Count	P_value
UP_KW_PTM	Acetylation	4	$1.5E^{-2}$
GOTERM_CC_DIRECT	nucleus	3	$2.1E^{-1}$
GOTERM_CC_DIRECT	Protein binding	3	$7.4E^{-1}$

Uncropped western blots.

Uncropped western blots Figure 3:
(HSP701A)

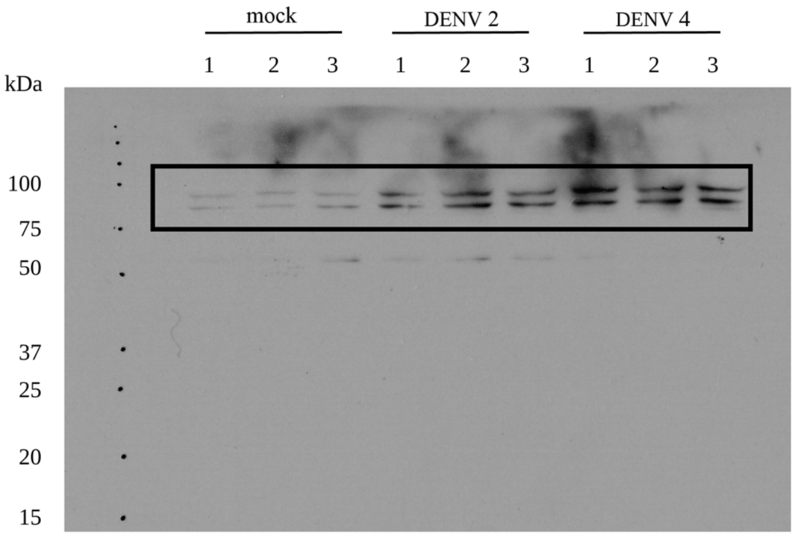

Uncropped western blots Figure 3:
(GAPDH for HSP701A)

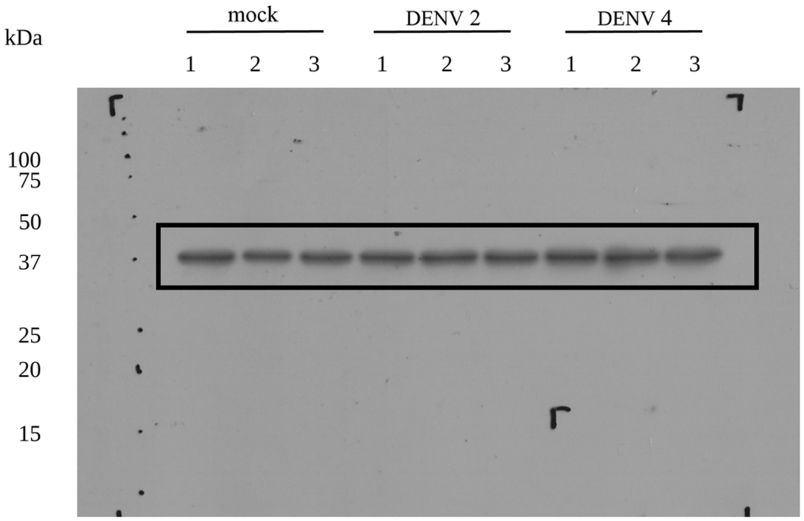

Uncropped western blots Figure 3:
(PHB 1)

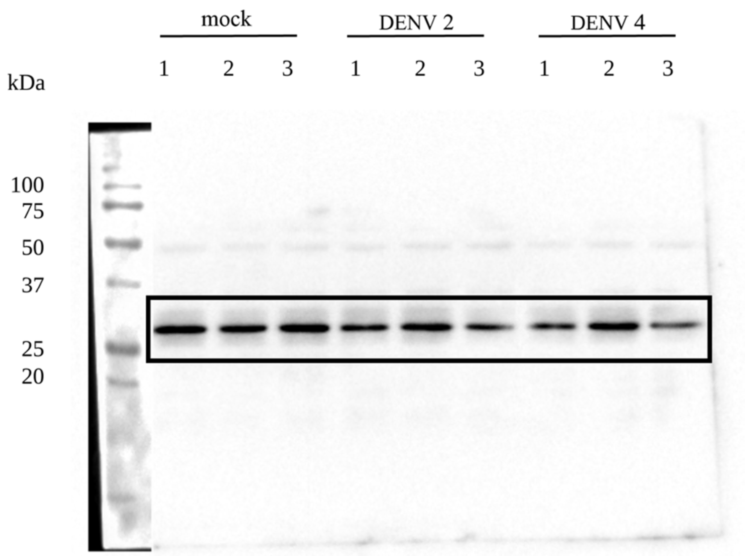

Uncropped western blots Figure 3:
(GAPDH for PHB1)

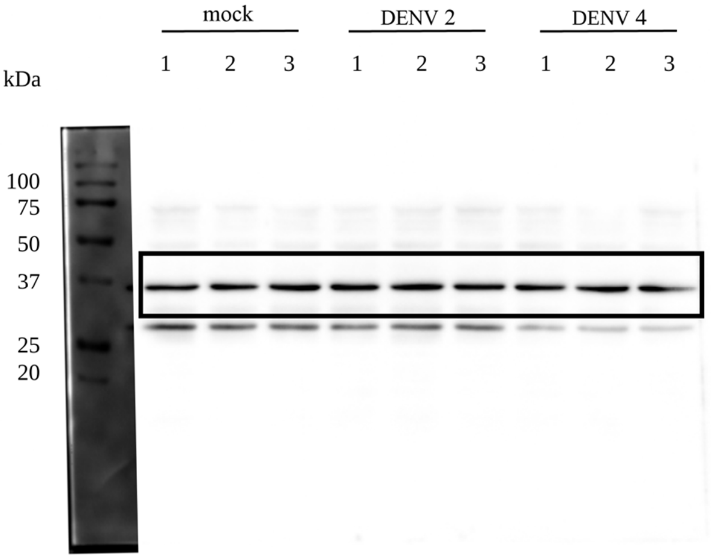

Uncropped western blots Figure 3:
(DENV E)

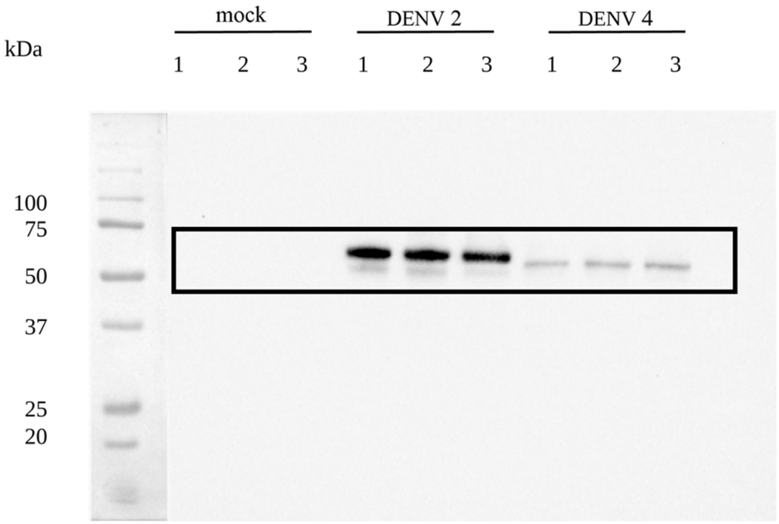

Uncropped western blots Figure 3:
(HSP90)

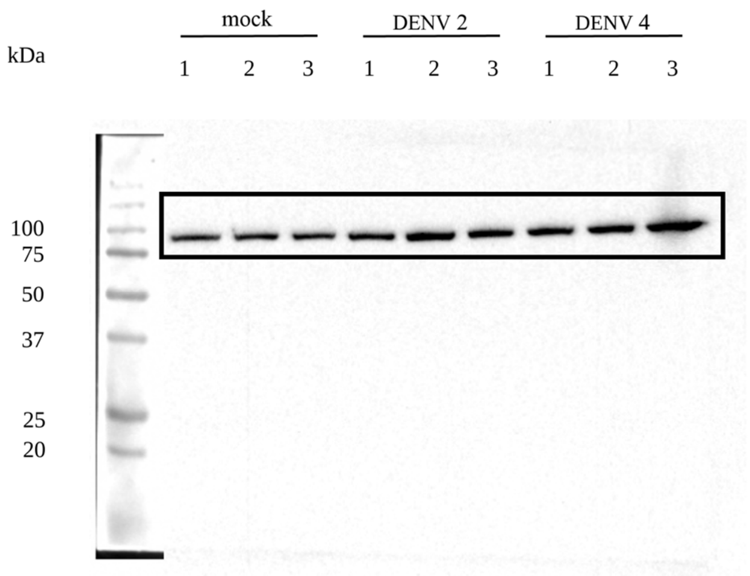

Uncropped western blots Figure 3:
(STIP1)

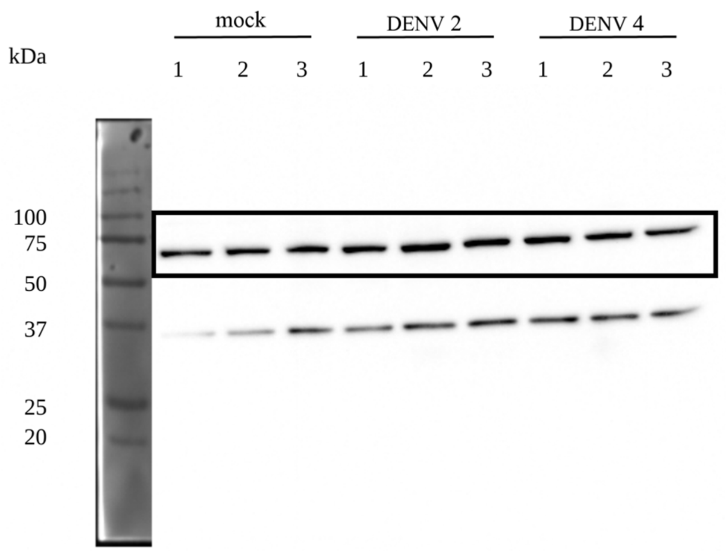

Uncropped western blots Figure 3:
(PPA1)

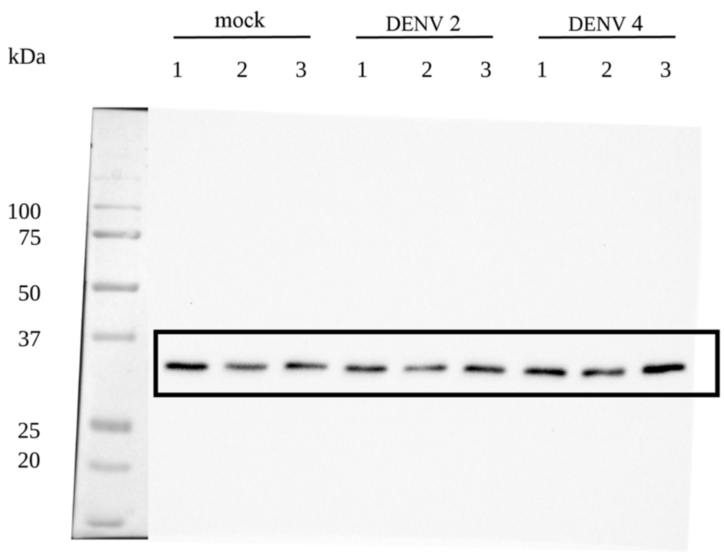

Uncropped western blots Figure 3:
(GAPDH)

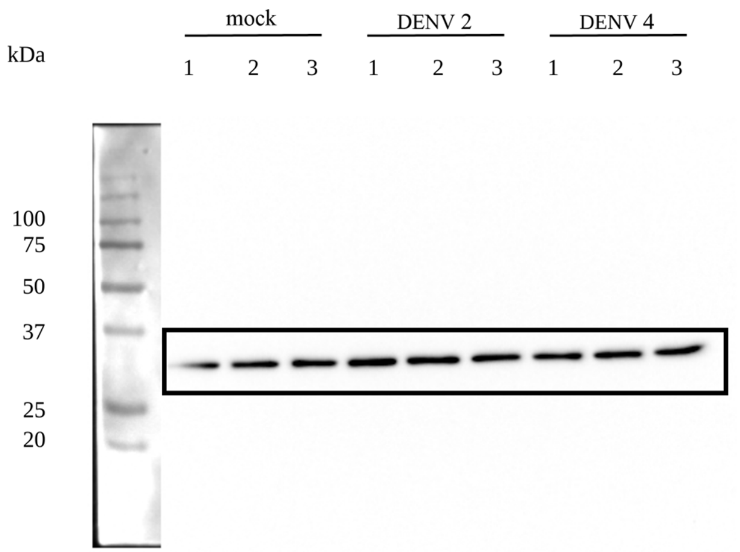

Uncropped western blots Figure 4A:
(PHB1)

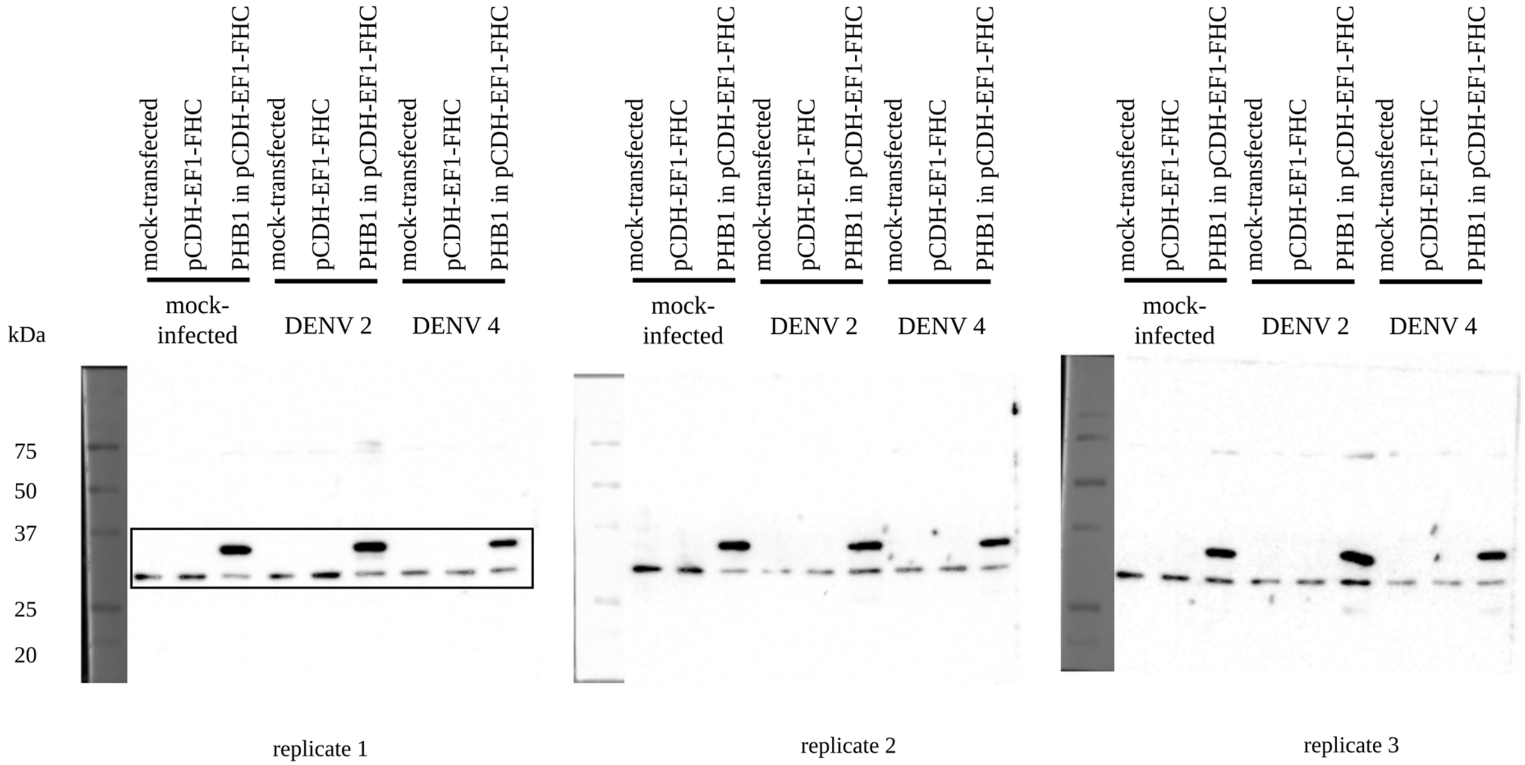

Uncropped western blots Figure 4A:
(DENV E)

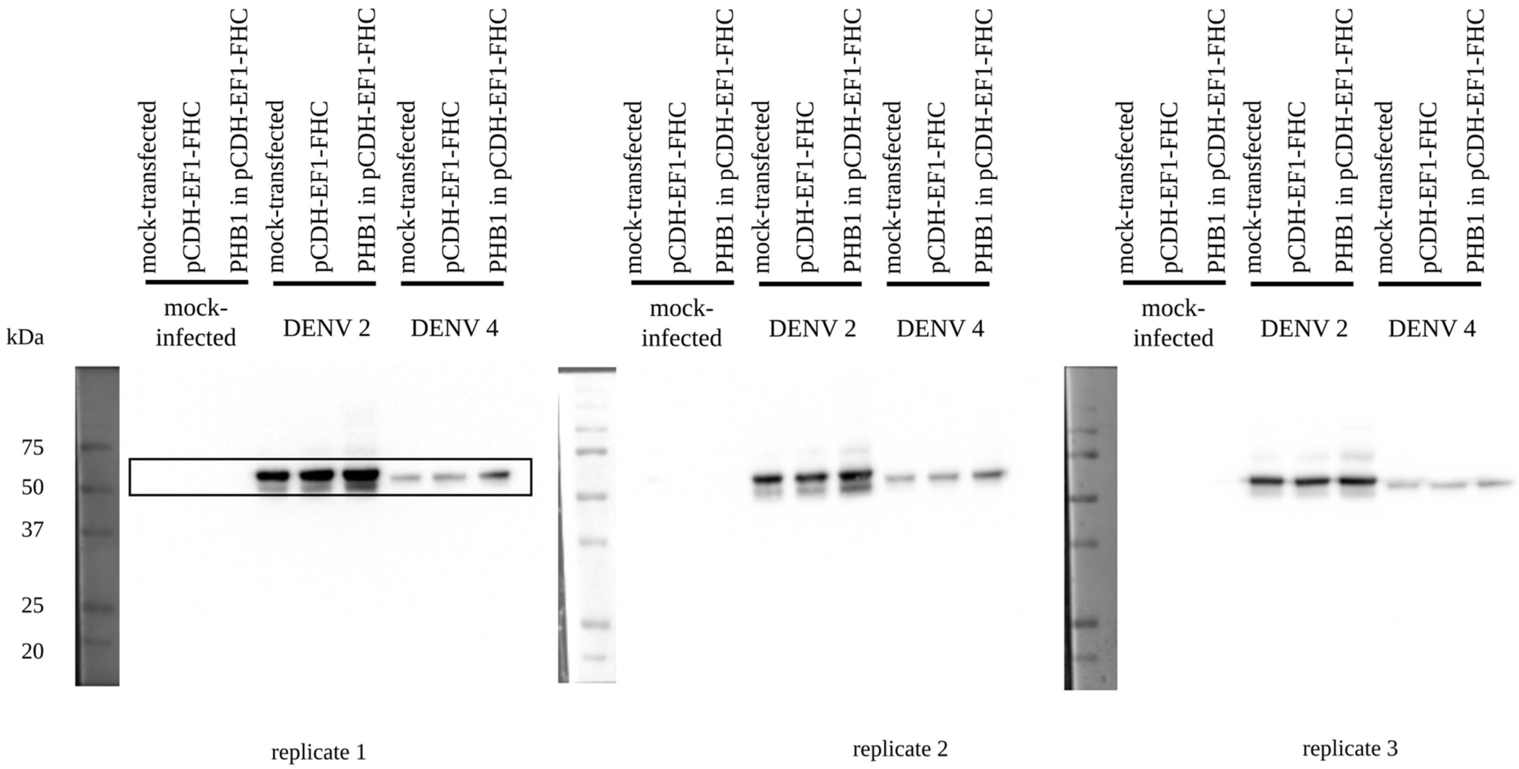

Uncropped western blots Figure 4A:
(DENV 2 NS3)

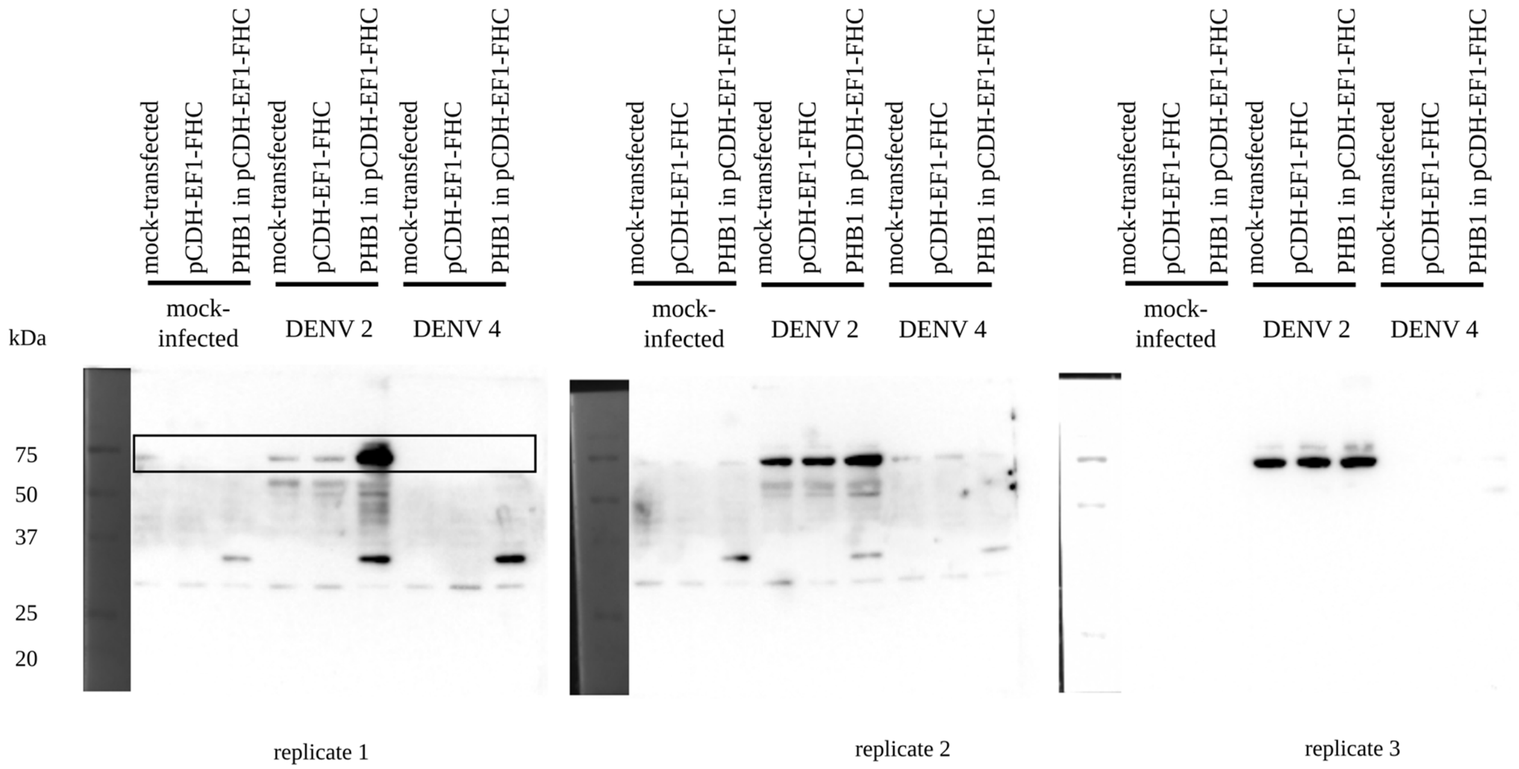

Uncropped western blots Figure 4A:
(Actin)

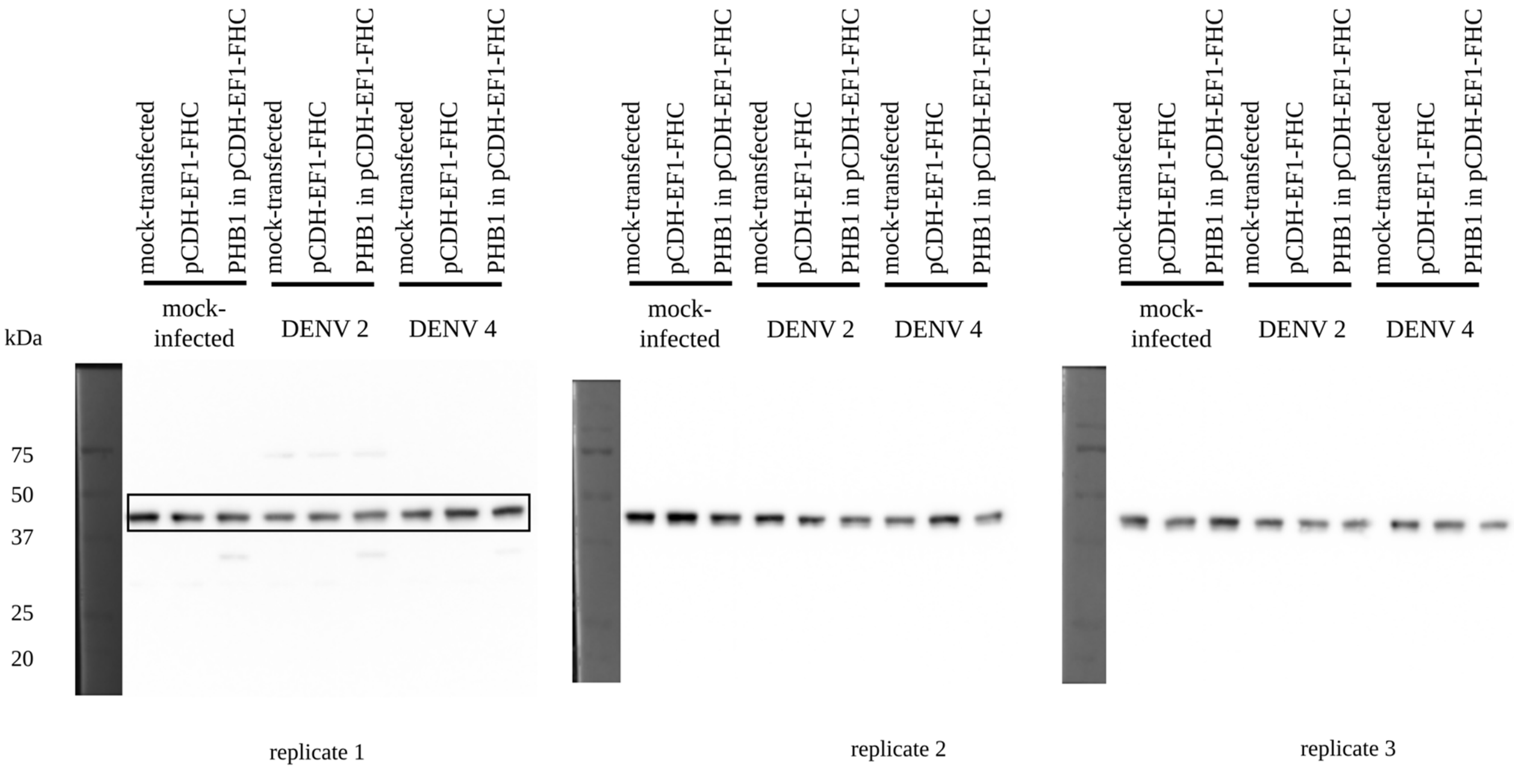

Uncropped western blots Figure 5C:
(PHB1)

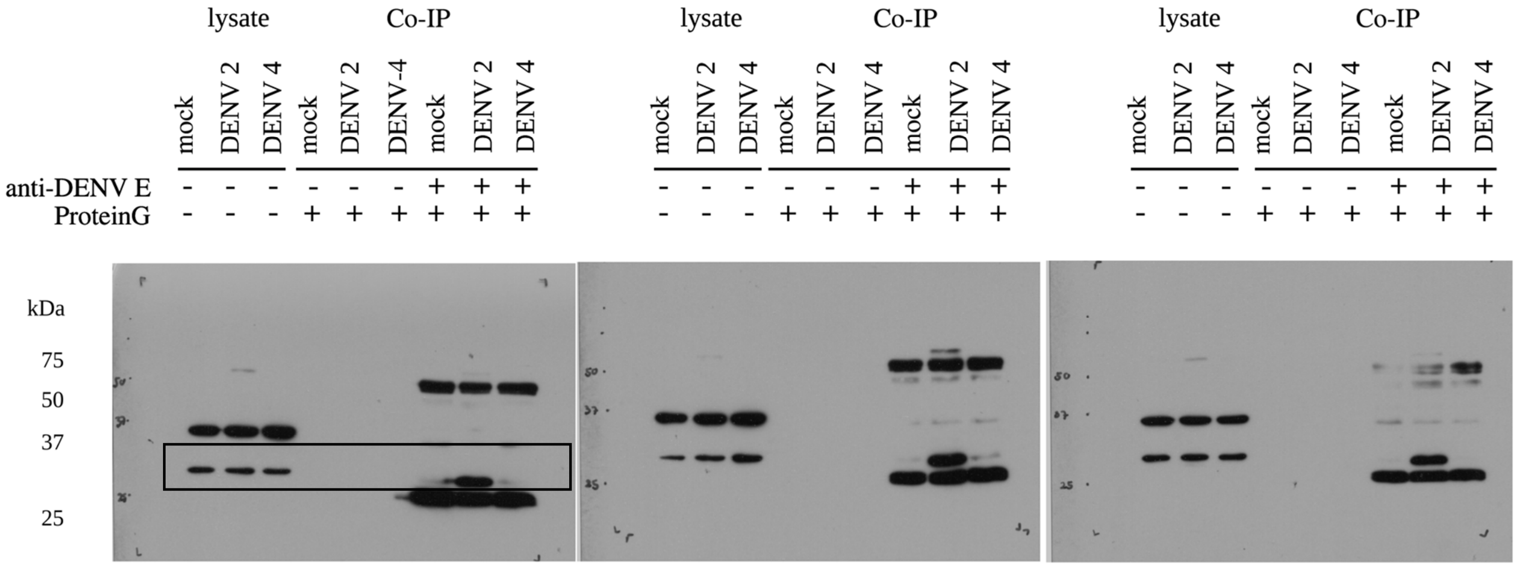

replicate 1

replicate 2

replicate 3

Uncropped western blots Figure 5C:
(DENV E)

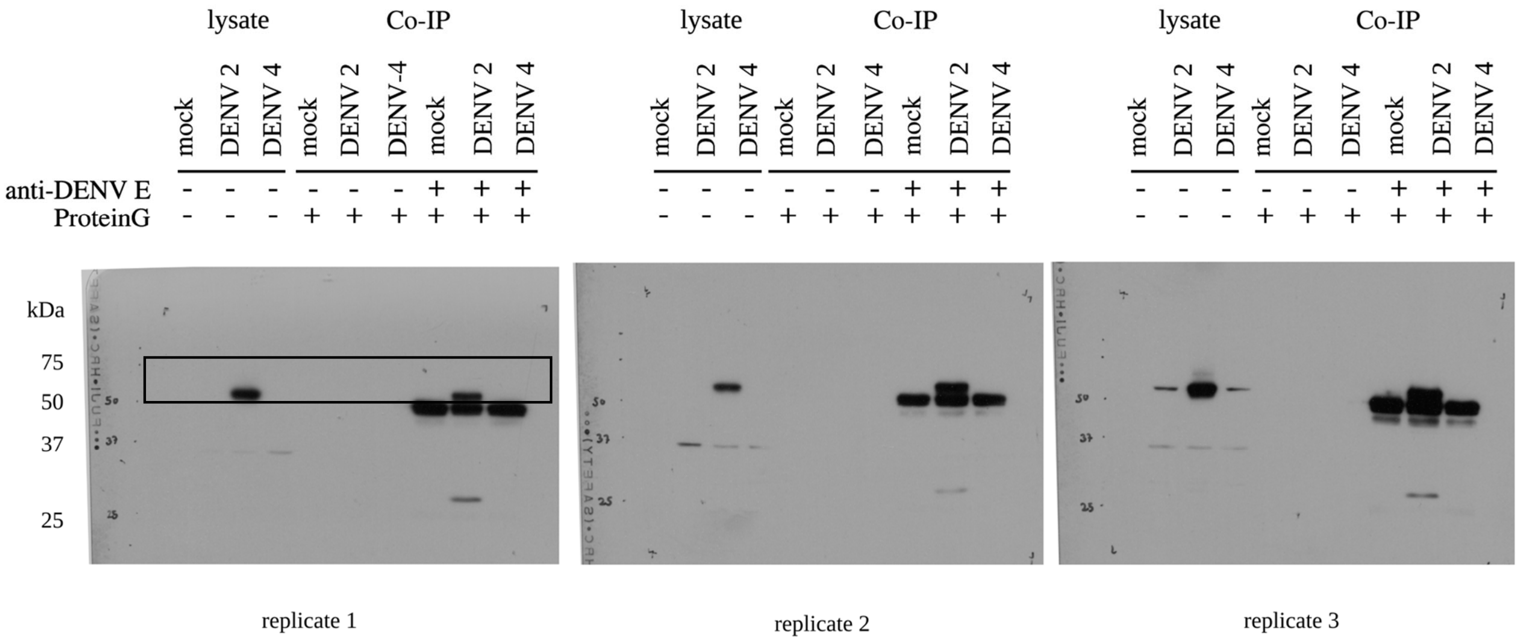

Supplement: Supplementary file 1 — Supplementary Information. [file 41598_2024_57930_MOESM1_ESM.pdf]
